# A Replicable and Generalizable Neuroimaging‐Based Indicator of Pain Sensitivity Across Individuals

**DOI:** 10.1002/advs.202503373

**Published:** 2025-09-05

**Authors:** Li‐Bo Zhang, Xue‐Jing Lu, Hui‐Juan Zhang, Zhao‐Xing Wei, Ya‐Zhuo Kong, Yi‐Heng Tu, Gian Domenico Iannetti, Li Hu

**Affiliations:** ^1^ State Key Laboratory of Cognitive Science and Mental Health Institute of Psychology Chinese Academy of Sciences Beijing 100101 China; ^2^ Department of Psychology University of Chinese Academy of Sciences Beijing 100049 China; ^3^ Neuroscience and Behaviour Laboratory Italian Institute of Technology Rome 00161 Italy; ^4^ Department of Neuroscience Physiology and Pharmacology University College London London WC1E 6BT UK

**Keywords:** functional magnetic resonance imaging (fMRI), individual differences, machine learning, neural indicator, pain sensitivity, sample size

## Abstract

Revealing the neural underpinnings of pain sensitivity is crucial for understanding how the brain encodes individual differences in pain and advancing personalized pain treatments. Here, six large and diverse functional magnetic resonance imaging (fMRI) datasets (total N = 1046) are leveraged to uncover the neural mechanisms of pain sensitivity. Replicable and generalizable correlations are found between nociceptive‐evoked fMRI responses and pain sensitivity for laser heat, contact heat, and mechanical pains. These fMRI responses correlate more strongly with pain sensitivity than with tactile, auditory, and visual sensitivity. Moreover, a machine learning model is developed that accurately predicts not only pain sensitivity (r = 0.20∼0.56, ps < 0.05) but also analgesic effects of different treatments in healthy individuals (r = 0.17∼0.25, ps < 0.05). Notably, these findings are influenced considerably by sample sizes, requiring >200 for univariate whole brain correlation analysis and >150 for multivariate machine learning modeling. Altogether, this study demonstrates that fMRI activations encode pain sensitivity across various types of pain, thus facilitating interpretations of subjective pain reports and promoting more mechanistically informed investigations into pain physiology.

## Graphical abstract

Humans differ in their sensitivity to pain. With six large and diverse fMRI datasets (total N = 1046), this study finds that such individual differences in pain sensitivity can be tracked by fMRI responses to painful stimuli. A highly generalizable machine learning model is further built to predict pain sensitivity across all datasets and analgesic effects of different treatments.

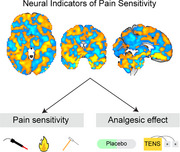

## 1 Introduction

Individuals differ in their sensitivity to pain, which is generally operationalized as individual differences in the pain detection threshold, pain tolerance threshold, or simply the mean intensity ratings of a series of identical painful stimuli.^[^ ^1^ ^]^ A noticeable observation is the considerable inter‐individual variability in pain sensitivity.^[^ ^2^ ^]^ The same nociceptive stimulus may be intolerably painful for someone, yet barely perceivable by another. What neural activity encodes such dramatic variability of pain sensitivity? This is a fundamental question in pain neuroscience. Understanding this will provide objective neurological indicators of pain sensitivity,^[^ ^3^, ^4^, ^5^ ^]^ and support mechanistically informed investigations of pain by enabling detailed mapping between specific brain regions or pathways underlying pain. Furthermore, neural indicators of pain sensitivity may also aid in screening high‐risk individuals for chronic pain and developing individualized pain treatment strategies,^[^ ^3^, ^6^, ^7^ ^]^ since high pain sensitivity is a risk factor for chronic pain.^[^ ^1^, ^8^, ^9^ ^]^

Numerous previous studies have attempted to find neural underpinnings of pain sensitivity with non‐invasive neuroimaging techniques like functional magnetic resonance imaging (fMRI).^[^ ^10^, ^11^, ^12^, ^13^, ^14^, ^15^ ^]^ However, three key issues remain unresolved as of now. First and foremost, it is still subject to heated debate whether nociceptive‐evoked brain activations can encode pain sensitivity, that is, whether brain activations can serve as neural indicators of pain sensitivity. Painful stimuli consistently activate a series of brain areas such as the primary somatosensory cortex (S1), secondary somatosensory cortex (S2), anterior cingulate cortex (ACC), insula, thalamus, and prefrontal cortex (PFC).^[^ ^16^, ^17^ ^]^ An early small (N = 17) but seminal study linked larger nociceptive‐evoked blood oxygen level dependent (BOLD) responses in the S1, ACC, and PFC with higher pain sensitivity.^[^ ^10^ ^]^ Yet, a recent study with a large sample size (N = 101) found that no single brain area significantly correlated with pain sensitivity.^[^ ^11^ ^]^ With a slightly larger sample size (N = 124), another recent study observed weak but significant correlations between pain sensitivity and nociceptive‐evoked BOLD responses in the S1, thalamus, supplementary motor area, and periaqueductal gray (PAG).^[^ ^18^ ^]^ These inconsistent findings may exemplify the replication crisis in empirical sciences.^[^ ^19^, ^20^, ^21^, ^22^ ^]^ One critical cause of non‐replicability is sample size.^[^ ^23^ ^]^ Recent studies suggest that a sample size of around 100 in functional neuroimaging studies may be insufficient to reveal the association between BOLD responses and behaviors at the inter‐individual level,^[^ ^24^, ^25^ ^]^ especially when the true correlation is small.^[^ ^18^, ^26^ ^]^

Another unresolved issue is whether nociceptive‐evoked brain responses selectively track pain sensitivity rather than modality‐independent stimulus factors.^[^ ^5^ ^]^ Previous studies have shown that nociceptive‐evoked neural responses can be evoked by nonpainful but equally salient tactile, auditory, and visual stimuli,^[^ ^27^, ^28^, ^29^, ^30^ ^]^ implying that these responses are not pain selective. However, recent studies have found that an area in the posterior parietal operculum responded to painful stimuli rather than salience‐matched auditory stimuli,^[^ ^31^ ^]^ and that part of the cingulate gyrus, precuneus, supplementary motor area, dorsolateral and medial part of superior frontal gyrus showed greater activities to pain instead of tactile, auditory, or visual stimuli.^[^ ^32^ ^]^ Furthermore, brain activity patterns in the medial‐dorsal thalamus preferentially encoded painful rather than nonpainful stimuli,^[^ ^33^ ^]^ and some voxels in the brainstem, thalamus, insula, anterior and middle cingulate cortex, and supplementary motor area consistently emerged in the models distinguishing painful stimuli from intensity‐ or salience‐matched tactile stimuli.^[^ ^34^ ^]^ These studies suggest potentially preferential encoding of pain in some brain regions. Notably, these studies have mainly focused on whether nociceptive‐evoked brain responses selectively track pain experience at the within‐individual level. Rarely have previous studies quantitatively examined whether certain brain region or activity pattern correlates with individual differences in pain and other modalities alike at the between‐individual level. In other words, it remains unclear whether there are selective neural indicators of individual differences in pain sensitivity.

The third issue concerns the generalizability of neural indicators of pain sensitivity. Pain can be induced by various types of nociceptive stimuli such as heat and mechanical pressure.^[^ ^35^ ^]^ An ideal neural indicator of pain sensitivity should be generalizable in terms of pain types. However, few studies have employed more than one type of nociceptive stimuli, even though the neural processing of different types of nociceptive stimuli can differ.^[^ ^36^ ^]^ For example, a recent study has shown that heat pain activates the precentral gyrus, pontine reticular nucleus, and dorsal posterior insula more than pressure pain does, whereas pressure pain leads to greater activation in the primary somatosensory cortex and bilateral superior parietal lobules compared with heat pain.^[^ ^37^ ^]^ It is thus of particular interest to develop generalizable neural indicators of pain sensitivity. Such neural indicators are less likely to be influenced by the specific peculiarities of individual pain types, instead capturing the common element shared across all pain types: the perceptual experience of pain.

**Figure 1:**
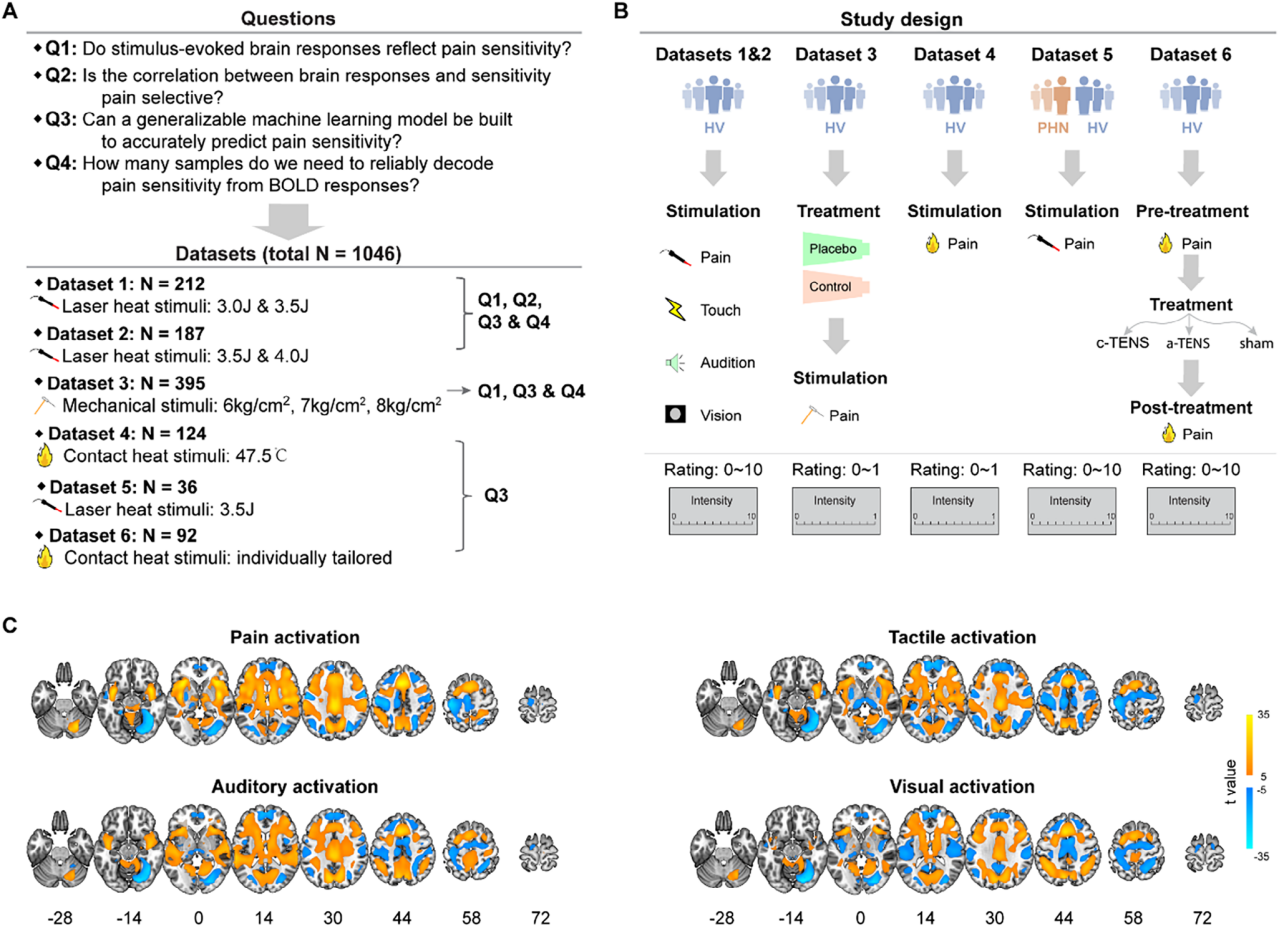
Study overview and brain activations responsive to painful and nonpainful stimuli. A) Key questions and datasets used to answer these questions. Using six datasets with laser heat pain, mechanical pain, and contact heat pain stimuli, we examined the correlation between nociceptive‐evoked BOLD responses and pain sensitivity, tested pain selectivity of this correlation, built generalizable machine learning models to predict pain sensitivity, and explored sample sizes needed to reliably reveal the correlation between BOLD responses and pain sensitivity. B) Study design of six datasets. Datasets 1, 2, 3, 4, and 6 only recruited healthy volunteers, and Dataset 5 recruited both postherpetic neuralgia patients and healthy volunteers. In all six datasets, participants received sensory stimuli and rated the perceived intensity with a rating scale (from 0 to 10 or from 0 to 1). In Datasets 3 and 6, participants also received treatments (placebo in Dataset 3 and transcutaneous electrical nerve stimulation [TENS] in Dataset 6) to relieve pain. C) Brain areas activated by painful and nonpainful stimuli in Datasets 1 and 2. Painful, tactile, auditory, and visual stimuli activated largely overlapped brain areas. HV: healthy volunteers; PHN: postherpetic neuralgia patients; c‐TENS: conventional TENS; a‐TENS: acupuncture‐like TENS. One‐sample t tests (N = 399) were conducted in (C).

To address these issues, we leveraged six ethnically and culturally diverse fMRI datasets (total N = 1046) from three countries (China, US, and South Korea), where healthy (Datasets 1–4, and 6) or chronic pain participants (Dataset 5) received various types of painful stimuli: laser heat pain in Datasets 1, 2, and 5,^[^ ^14^, ^38^ ^]^ mechanical pain in Dataset 3,^[^ ^39^ ^]^ contact heat pain in Datasets 4^[^ ^18^ ^]^ and 6^[^ ^40^ ^]^ (see Table S1, Supporting Information for a brief summary of each dataset). We aimed to answer four key questions (**Figure** **1**A): 1) Do nociceptive‐evoked BOLD responses reflect pain sensitivity across individuals? 2) If so, is this correlation selective to the pain modality? 3) Can we develop a generalizable machine learning (ML) model that accurately predicts pain sensitivity? 4) Which sample size is needed to decode pain sensitivity from BOLD responses? First, we correlated nociceptive‐evoked BOLD responses with pain sensitivity and tested the replicability and generalizability of the correlation with Datasets 1–3 (N = 794). Subsequently, we examined whether the correlation between BOLD responses and sensory sensitivity differs between pain and nonpain modalities with Datasets 1 and 2 (N = 399), where participants also received nonpainful tactile, auditory, and visual stimuli (Figure 1B). Next, we built a multivariate ML model, named the Neuroimaging‐based Indicator of Pain Sensitivity (NIPS) model, to predict pain sensitivity using nociceptive‐evoked BOLD responses. We evaluated NIPS's performance with Datasets 1 and 2, examined its generalizability across various pain types and diverse populations with three external datasets (i.e., Datasets 3, 4, and6, total N = 611), and tested its ability to predict pain reduction from different interventions in healthy individuals (i.e., Datasets 4 and 6, N = 487) and pain sensitivity in chronic pain patients (i.e., Dataset 5, N = 36). Finally, we systematically examined the influence of sample sizes on the ability to detect the univariate correlation between BOLD responses and pain sensitivity with 80% statistical power^[^ ^41^ ^]^ and achieve stable performance in multivariate predictive modeling.

## 2 Results

### 2.1 BOLD Responses Reflect Pain Sensitivity

**Figure 2:**
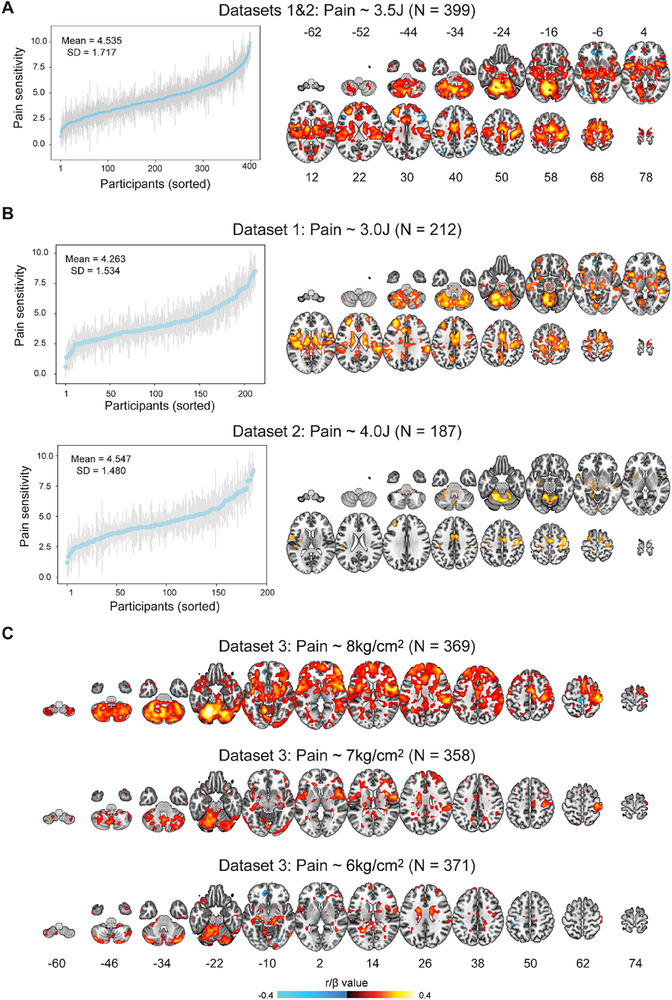
Correlations between nociceptive‐evoked BOLD responses and pain sensitivity. A) BOLD‐pain sensitivity correlations in the 3.5J condition in Datasets 1 and 2. Pain sensitivity varied considerably across individuals. This interindividual variability correlated with nociceptive‐evoked BOLD responses in a wide range of areas. B) Replication of the correlation in other conditions in Datasets 1 and 2. BOLD responses correlated with pain sensitivity in the 3.0 and 4.0J conditions. C) Replication of the correlation in Dataset 3. BOLD responses were associated with mechanical pain sensitivity in all conditions, demonstrating the replicability and generalizability of the correlation. Error bars in panels A and B stand for standard deviation (SD) of intensity ratings for each participant. Color bars indicate Pearson's r values in panels A and B, and standardized coefficients (β) for the fixed effect of BOLD responses in mixed effects models that considered observation dependence within families in panel C.

Dataset 1 delivered 10 laser heat pain stimuli at intensities of 3.0J and 3.5J (20 stimuli in total), and Dataset 2 delivered 10 stimuli at 3.5J and 4.0J (20 stimuli in total). To make full use of the large sample size, we pooled data from the 3.5J condition across both datasets (N = 399) and referred to it as the 3.5J condition. Pain sensitivity was quantified as the mean of the 10 ratings in a condition. As in previous studies,^[^ ^2^, ^11^, ^18^ ^]^ we observed considerable inter‐individual variability of pain sensitivity in the 3.5J condition even though the painful stimuli had identical physical intensity. Pain sensitivity ranged from 0.8 to 9.9 (mean ± standard deviation [SD]: 4.54 ± 1.72; **Figure** **2**A; and Table S4, Supporting Information), covering almost the entire range of the 0 (“‘no sensation”’) ∼ 10 (“the strongest sensation imaginable”) rating scale. Painful stimuli also activated a wide range of typically pain‐related brain areas, including the S1, S2, ACC, insula, and thalamus (Figure 1C).

Importantly, we found significant Pearson's correlations between nociceptive‐evoked BOLD responses and pain sensitivity across individuals (Figure 2A, see the unthresholded map in https://osf.io/y2n34/; *p(FDR)* < 0.05, the same hereafter). Positive correlations were found in a wide range of areas, including but not limited to the S1, S2, ACC, insula, thalamus, and cerebellum. Negative correlations with pain sensitivity were only observed in a few areas including part of the middle frontal gyrus, inferior parietal gyrus, and middle temporal gyrus. The absolute magnitude of negative correlations was generally smaller than those of positive correlations. To test the analytical robustness of this finding,^[^ ^42^ ^]^ we also conducted Spearman's rank correlation analysis. A similar significant correlation pattern was found (Figure S1A, Supporting Information). In the analyses above, we accounted for head motion with the classical six motion parameters. To further control the potential influence of head motion, we regressed out from the BOLD signals the six motion parameters, their first derivatives, and squares of the motion parameters and first derivatives.^[^ ^43^ ^]^ Still, the significant correlation pattern resembled that of our original finding (Figure S1B, Supporting Information), suggesting a negligible influence of head motion on our finding.

In the foregoing analyses, we pooled the 3.5J condition in Datasets 1 and 2 since these datasets were assumed to only differ in pain sensitivity of participants and laser stimulation parameters based on the data collection process. Indeed, participants in Dataset 1 reported greater pain ratings to the 3.5J stimulus than those in Dataset 2 (t(397) = 10.637, *p* < 0.0001, Cohen's d = 1.067), and the sex ratio (χ^2^(1) = 3.434, *p* = 0.064) and age (t(395) = 1.048, *p* = 0.295, Cohen's d = 0.105) did not differ in these two datasets. However, there could be some unknown systematic differences or site effects between the two datasets. We therefore conducted partial correlation analysis between BOLD responses and pain sensitivity while controlling for dataset, sex, and age (see Experimental Section for details). Removing the influence of these variables had some effect on the magnitude of the correlation between BOLD responses and pain sensitivity, but the general pattern of the partial correlation results remained highly similar to the original correlation results (see Figure S2A, Supporting Information), demonstrating that dataset differences between Datasets 1 and 2 had no substantial effect on our findings.

We then examined whether our finding could be replicated with the same dataset and generalized to other datasets. In both the 3.0J condition in Dataset 1 and the 4.0J condition in Dataset 2, significant correlations were present in a series of brain areas (Figure 2B). However, fewer areas showed significant correlations in these two conditions, which could be associated with their sample sizes being only half of the pooled 3.5J condition (≈200 vs ≈400), thereby reducing the statistical power to detect true effects. Note also that fewer areas survived correction in the 4.0J condition than in the 3.0J condition. The pain ratings were slightly but not significantly higher in the 4.0J condition than in the 3.0J condition (t(397) = 1.872, *p* = 0.062, Cohen's d = 0.188), and the range of ratings was also not significantly different (4.0J condition range: 1.200 to 8.800, 3.0J condition range: 0.600 to 8.500, bootstrapping *p* = 0.628). These findings suggest that low correlations in the 4.0J condition were unlikely to be caused by rating differences in these two conditions, but may be related to the fact that Dataset 2 included participants with lower pain sensitivity than Dataset 1.

Dataset 3 delivered mechanical pain stimuli of three intensity levels to participants (i.e., 8, 7, and 6 kg cm^−2^). We also found significant associations between nociceptive‐evoked BOLD responses and pain sensitivity in all three intensity conditions (Figure 2C). Notably, the largest correlation coefficient was consistently around 0.4 in Datasets 1–3. Fewer areas showed significant correlations as the physical intensity decreased. One plausible explanation could be that painful stimuli of low intensity, such as 6 kg cm^−2^, might not consistently induce pain sensation, potentially resulting in a poor signal‐to‐noise ratio of the BOLD responses. Indeed, in the 6 kg cm^−2^ condition, 101 participants (27.2%) had a mean pain rating <0.014, which was defined as “barely detectable pain”. Similar to the 3.5J condition in Datasets 1 and 2, controlling for the influence of sex and age had no substantial effect on the results in Dataset 3 (Figure S3, Supporting Information). Notably, in agreement with laser heat pain in Datasets 1 and 2 and mechanical pain in Dataset 3, a previous study analyzing Dataset 4, where contact heat pain stimuli were delivered, also observed significant correlations between BOLD responses and pain sensitivity.^[^ ^18^ ^]^ Consistent findings in these datasets demonstrate not only the replicability of the correlation between BOLD responses and pain sensitivity, but also the generalizability of the correlation across different types of painful stimuli in ethnically and culturally diverse populations. To directly test whether classical pain‐related areas encode pain sensitivity, we extracted mean signals from the S1, S2, ACC, insula, thalamus, and cerebellum in Datasets 1–3 using anatomically defined masks. Significant correlations were observed in most of these regions of interest (ROI) except when the physical intensity was low (i.e., 6 kg cm^−2^) in Dataset 3 (Tables S5 and S6, Supporting Information). To assess the overlapping of pain‐encoding areas in our datasets, we computed the Dice coefficient between Figure 2A (3.5J condition) and Figure C (8 kg cm^−2^ condition). These two maps had a Dice coefficient of 0.60, suggesting ≈60% of overlapping in significant areas (see also the conjunction map in Figure S4A, Supporting Information).

**Figure 3:**
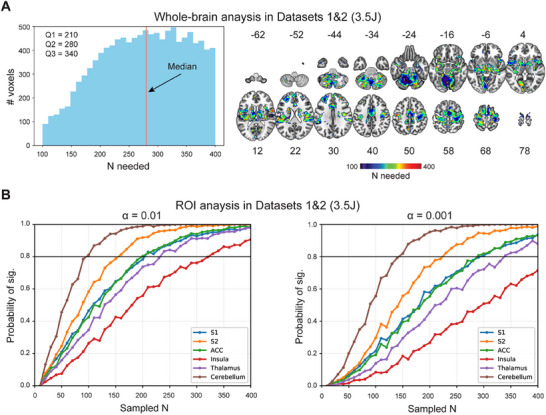
Influence of sample sizes on the detectability of the BOLD responses‐pain sensitivity correlation. A) Sample sizes needed to detect correlation with a probability ≥ 0.8 in whole brain analysis in Datasets 1 and 2. Different sample sizes were needed to reliably detect the correlations in different voxels, and a sample size of 280 was needed to detect half of the correlations. B) Sample sizes needed to detect correlation in ROI analysis in Datasets 1 and 2. In ROI analysis, large sample sizes were still needed to reliably detect significant correlations. Q1: first quartile; Q2: second quartile; Q3: third quartile. Bootstrapping tests were conducted with various sample sizes.

Given the inconsistent findings in previous studies with smaller sample sizes,^[^ ^10^, ^11^, ^18^ ^]^ we systematically investigated the potential influence of sample size on the BOLD responses‐pain sensitivity correlation in the 3.5J condition from Datasets 1 and 2. Briefly, we sampled with replacement 100–400 participants (in steps of 10) from Datasets 1 and 2, repeated the process 100 times, and estimated the sample size needed to detect a significant correlation in every voxel with a probability ≥0.8 (see Experimental Section for details).^[^ ^41^ ^]^ The sample size needed for different voxels varied greatly across the brain (**Figure** **3**A). For voxels that showed significant correlations, the median sample size needed was 280, with an SD = 78 (Figure 3A). To specifically focus on the pain‐related ROIs, we also estimated the probability of obtaining significant correlations for the S1, S2, ACC, insula, thalamus, and cerebellum (Figure 3B). With α = 0.01, the cerebellum required the smallest sample size (≈100) to reach significance with a probability of 0.8; with α = 0.001, the cerebellum required a sample size of ≈150. These findings demonstrate that the sample size had an enormous influence on the ability to detect significant correlations between BOLD responses and pain sensitivity.

### 2.2 BOLD Responses Differentially Reflect Pain Sensitivity and Nonpain Sensitivity

**Figure 4:**
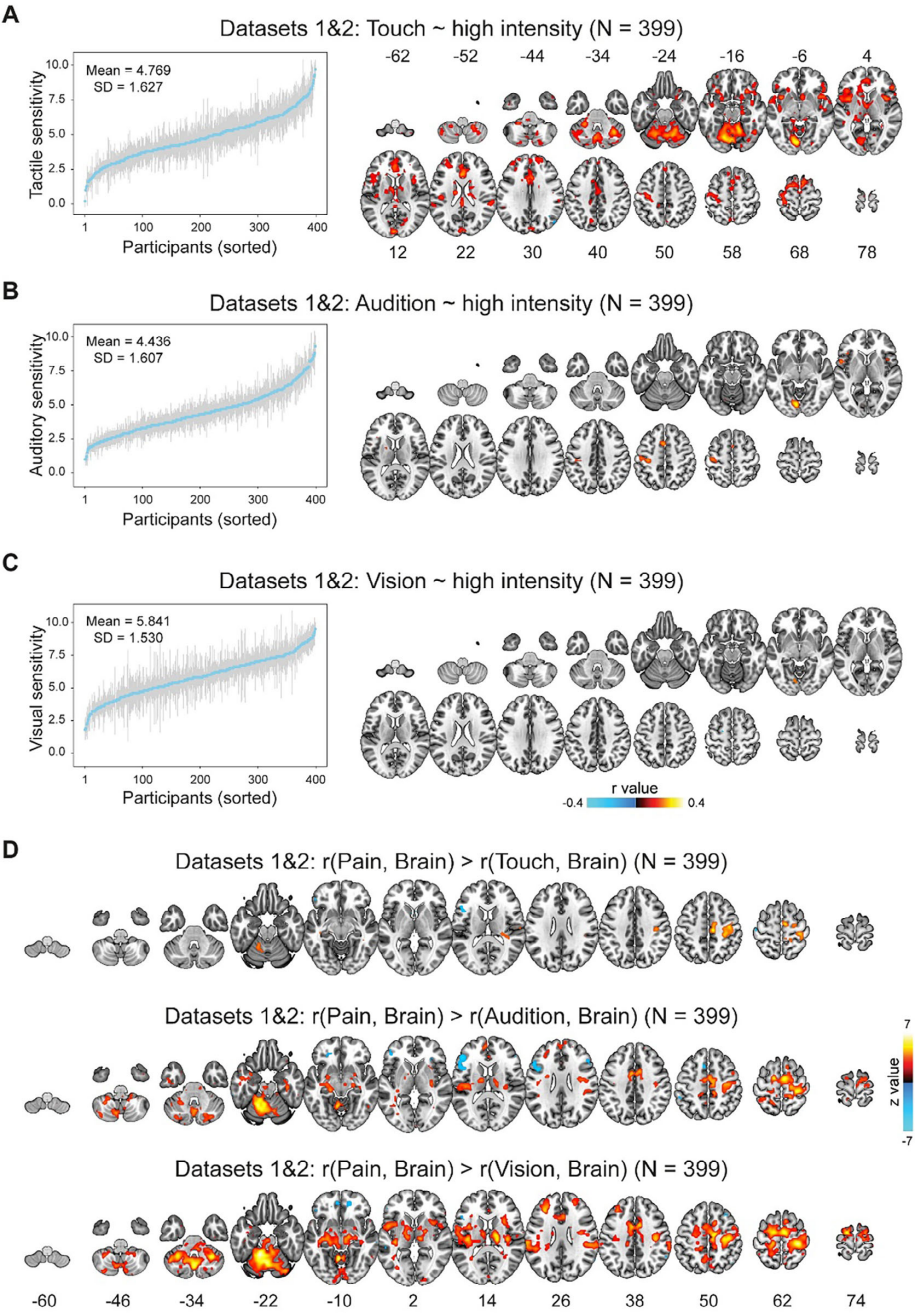
Correlations between BOLD responses and sensory sensitivity in nonpain modalities. A–C) BOLD responses‐sensory sensitivity correlation in the high intensity condition in Datasets 1 and 2 for touch (A), audition (B), and vision (C). Inter‐individual variability of tactile sensitivity correlated with BOLD responses in many areas, while only a few areas showed correlation with auditory and visual sensitivity. D) Comparisons of BOLD‐sensitivity correlation between pain and nonpain modalities. Direct comparisons of correlation coefficients showed larger correlations for pain than for touch, audition, and vision. Error bars in (A–C) stand for standard deviation (SD) of intensity ratings for each participant. In (A–C), Pearson's correlation analyses were conducted; in (D), Steiger's z tests were conducted.

Having shown that BOLD responses reflect pain sensitivity, we then examined in the full Datasets 1 and 2 whether the correlation is selective to the pain modality by correlating tactile, auditory, and visual stimuli‐evoked BOLD responses with the corresponding sensory sensitivity. In agreement with previous findings,^[^ ^28^ ^]^ brain regions activated by tactile, auditory, and visual stimuli were highly similar to those activated by painful stimuli (Figure 1C). However, Pearson's correlational results exhibited distinct patterns across modalities. BOLD responses evoked by high intensity tactile stimuli correlated significantly with tactile sensitivity in several brain areas (**Figure** **4**A), while significant correlations only occurred in some small and scattered brain areas for high intensity auditory and visual stimuli (Figure 4B,C). These results remained largely the same if Spearman's rho was computed rather than Pearson's r (Figure S5, Supporting Information), or the effect of dataset, sex, and age were controlled for (Figure 2B–D). Notably, these nonpain sensitivity‐encoding areas had limited overlapping with pain sensitivity‐encoding areas (for pain (Figure 2A) vs and touch (Figure 4A), the Dice coefficient was 0.18; for pain versus audition (Figure 4B), the Dice coefficient was 0.01; for pain versus vision (Figure 4C), the Dice coefficient was 0.006; also see conjunction maps in Figure S4B–D, Supporting Information). In contrast to the high intensity condition, only a few brain areas showed significant positive correlations in the low intensity conditions for all three nonpain modalities (Figure S6, Supporting Information). As the stimulus intensity seemed to exert substantial influences on the results, we focused on the high intensity conditions hereafter. We then directly compared correlation coefficients between pain and nonpain modalities. For the pain versus touch comparison, significant differences in correlation coefficients were observed in some small areas, notably in the S1, precentral gyrus, and supplementary motor area (Figure 4D). For pain versus audition and pain versus vision comparisons, significant differences were found in much more areas, covering typical pain‐related areas, such as the S1, S2, ACC, insula, and thalamus (Figure 4D).

### 2.3 NIPS Accurately Predicts Sensitivity to Various Pains in Diverse Populations

**Figure 5:**
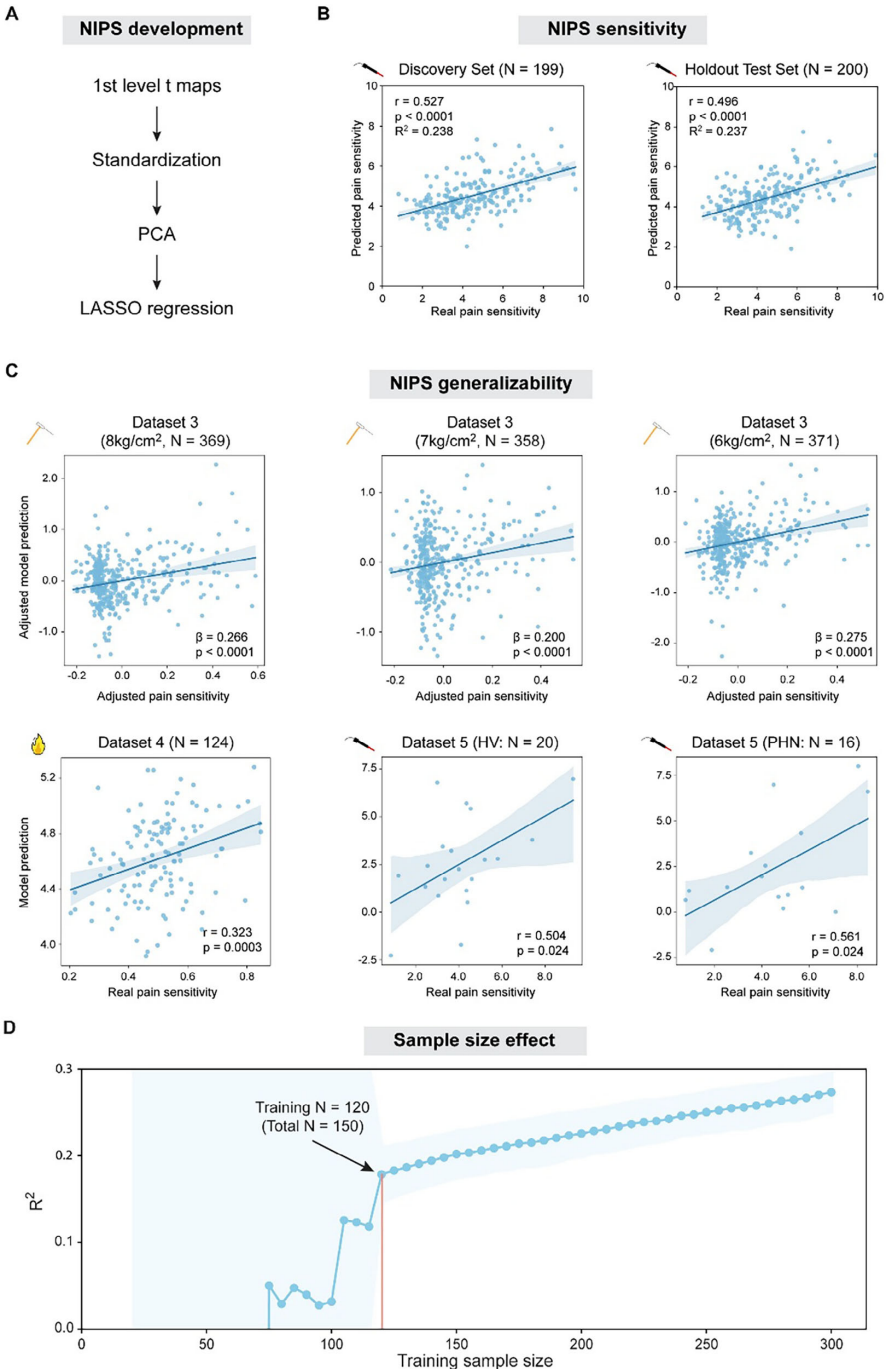
Development of NIPS and its performance. A) Development of NIPS. We used first level t maps for nociceptive stimuli as features and built our model (NIPS) with LASSO‐PCR. B) Performance of NIPS in the Discovery Set and Holdout Test Set. In both datasets, NIPS showed good performance, explaining >20% of the variance of pain sensitivity. C) Generalizability of NIPS. Although developed with laser heat pain data, NIPS significantly predicted mechanical and contact heat pain sensitivity in external datasets. Furthermore, NIPS also predicted pain sensitivity in postherpetic neuralgia patients in Dataset 5. In Dataset 3 results, “adjusted” means that random effects of family were adjusted. β is the standardized coefficient in mixed effects models. Note that real and predicted values in other datasets were not adjusted since dependent observations were not present in these datasets. D) Effect of sample size on NIPS performance. NIPS's performance varied dramatically with small training sample sizes, but stabilized and continuously improved as training sample sizes exceeded 120. Shaded regions in (B) and (C) stand for 95% confidence intervals, and shaded regions in (D) represent standard deviation (SD) of R^2^. HV: healthy volunteers; PHN: postherpetic neuralgia patients.

We next developed a neuroimaging‐based ML model (i.e., NIPS) to predict pain sensitivity across individuals. We first split randomly the pooled 3.5J data in Datasets 1 and 2 into a Discovery Set (N = 199) and a Holdout Test Set (N = 200). To build the model, we used the least absolute shrinkage and selection operator‐based principal component regression (LASSO‐PCR; **Figure** **5**A). In the Discovery Set, NIPS accurately predicted pain sensitivity with its five‐fold cross‐validated performance as follows: r = 0.527, *p *< 0.0001, R^2^ = 0.238 (Figure 5B). NIPS also performed well in the Holdout Test Set: r = 0.496, *p* < 0.0001, R^2^ = 0.237 (Figure 5B). NIPS thus could explain >20% of the variance of pain sensitivity in both the Discovery Set and Holdout Test Set. Even though NIPS was built based on the data from the 3.5J condition in the Discovery Set, it was also able to predict pain sensitivity in the 3.0J condition (N = 101; r = 0.404, *p* < 0.0001, R^2^ = 0.146) and the 4.0J condition (N = 99; r = 0.489, *p* < 0.0001, R^2^ = 0.229) in the Holdout Test Set. These findings demonstrate that NIPS can accurately predict pain sensitivity, explaining ≈20% of the variance of pain sensitivity across individuals.

NIPS was developed using BOLD responses evoked by laser heat pain. To examine its generalizability, we then used it to predict mechanical pain sensitivity in Dataset 3 and contact heat pain sensitivity in Dataset 4. In Dataset 3, NIPS significantly predicted mechanical pain sensitivity in all three conditions: 1) the 8 kg cm^−2^ condition: β = 0.266, *p* < 0.0001; 2) the 7 kg cm^−2^ condition: β = 0.200, *p* < 0.0001; 3) the 6 kg cm^−2^ condition: β = 0.275, *p* < 0.0001 (Figure 5C). Likewise, NIPS significantly predicted contact heat pain sensitivity in Dataset 4: r = 0.323, *p* = 0.0003 (Figure 5C). Interestingly, this correlation was close to what the original authors of Dataset 4 observed (r = 0.252) in their own study, where a specific model was built to predict contact heat pain sensitivity.^[^ ^18^ ^]^ To test the applicability of NIPS in patient groups, we also applied it to a clinical dataset (i.e., Dataset 5). NIPS predicted pain sensitivity in both postherpetic neuralgia patients (r = 0.561, *p* = 0.024) and age‐ and sex‐matched healthy individuals (r = 0.504, *p* = 0.024) (Figure 5C). As in the univariate analysis, we also controlled for dataset, sex, and age, and found that the revised model still predicted pain sensitivity in Datasets 1–5 (Figure S7B,C, Supporting Information). Further analyses controlling for only dataset identity, sex, or age showed that controlling for dataset led to performance reduction similar to that observed when controlling for all three variables, while controlling for sex or age had little impact on the model performance (Figure S8, Supporting Information), suggesting that dataset heterogeneity is largely caused by dataset identity. Note that while Datasets 1–4 included young and middle‐aged adults, Dataset 5 recruited older adults as participants (mean age>60 years). Furthermore, these datasets also included participants from different ethnicity and cultural backgrounds. Taken together, these results suggest that NIPS is a generalizable neural indicator of pain sensitivity.

To examine the effect of sample size on the model performance, we also resampled data (training sample size: 20 to 300, in steps of 5) in the pooled 3.5J condition in Datasets 1 and 2. Note that five‐fold cross‐validation was adopted to assess the model performance. Therefore, for a sample of 100 participants, the training sample size (the abscissa in Figure 5D) is 80. When the training sample size was smaller than or equal to 70 (i.e., total sample size ≈88), NIPS's performance was very poor, even worse than a simple mean prediction (R^2^<0), and varied dramatically. When the training sample size was around 100 (i.e., 95, 100, or 105), the R^2^ of NIPS was ≈0.06. Interestingly, this estimate was very close to what Gim et al.^[^ ^18^ ^]^ found with a training sample size of 99 (model performance: r = 0.252, R^2^ = 0.064). Nevertheless, with such a sample size, NIPS's performance still varied substantially. The performance stabilized only after the training sample size reached 120 (i.e., total N = 150) and then R^2^ kept increasing with the training sample size. These findings suggest that a large sample size (>150) is needed to build a well‐performing pain prediction model.

While NIPS performed relatively well in our datasets, it explained only a small part of the variance of pain sensitivity. Since behavioral measures other than intensity ratings were available in Datasets 1 and 2, we explored whether incorporating them with fMRI responses could help build better predictive models. As a first step to build this composite model, we trained a model with behavioral measures including quantitative sensory testing and a series of questionnaires assessing psychological traits such as fear of pain, pain anxiety, and depression (see supplemental data file 1 for details). The behavior only model could also predict pain sensitivity: 1) Discovery Set: r = 0.447, *p* < 0.0001, R^2^ = 0.163; 2) Holdout Test Set: r = 0.356, *p* < 0.0001, R^2^ = 0.123 (Figure S9B, Supporting Information). More importantly, utilizing both fMRI responses and behavioral measures led to a better‐performing model: 1) Discovery Set: r = 0.620, *p* < 0.0001, R^2^ = 0.354; 2) Holdout Test Set: r = 0.606, *p* < 0.0001, R^2^ = 0.361 (Figure S9C, Supporting Information). The R^2^ of the fMRI + behavior model was roughly the sum of the fMRI only model and behavior only model. While the generalizability of this composite model could not be tested due to the unavailability of behavioral measures in other datasets, our result still illustrates a promising approach for constructing better composite models.

### 2.4 NIPS is a Distributed Pattern Predictive of Pain Reduction

**Figure 6:**
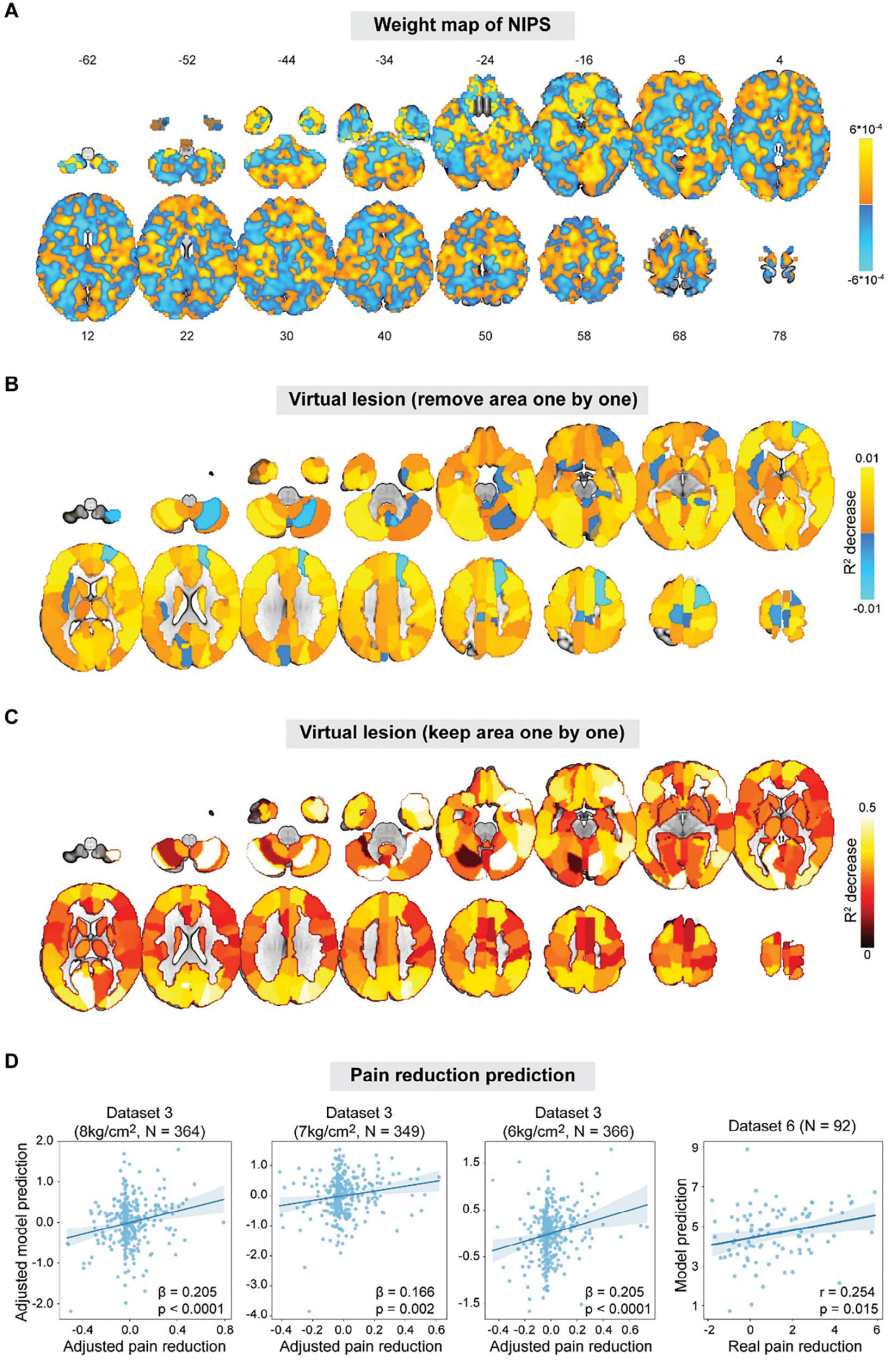
Region importance and pain reduction prediction of NIPS. A) Weight map of NIPS. Pain sensitivity predicting voxels are distributed over the entire brain. B) Region importance assessed by removing areas one by one in the AAL template. Removing only single brain region did not make NIPS's performance substantially worse, suggesting that a distributed network rather than isolated areas is responsible for representing pain sensitivity. C) Region importance assessed by keeping areas one by one in the AAL template. Since the R^2^ of NIPS was only ≈0.24, keeping only single brain region substantially impacted NIPS's performance, suggesting that regions work together to represent pain sensitivity. D) Prediction of pain reduction by NIPS in healthy individuals. In Datasets 3 and 6, NIPS significantly predicted pain reduction after pain treatments, suggesting NIPS could be related to pain modulation. In Dataset 3 results, “adjusted” means that random effects of family were adjusted. β is the standardized coefficient in mixed effects models. Note that real and predicted values in Dataset 6 were not adjusted since dependent observations were not present in this dataset. Shaded regions in (C) stand for 95% confidence intervals.

Having comprehensively tested NIPS's performance, we then attempted to understand what it represents. Its weight map showed a largely distributed pattern, with contribution from the entire the brain (**Figure** **6**A). To explore which areas were more important for NIPS, we parcellated the brain according to the Automatic Anatomical Labeling template,^[^ ^44^ ^]^ removed (i.e., “virtually lesioned”) each area, and refitted the predictive model without the corresponding area. Although the effect of removing areas was not uniform across the brain, none of the areas was practically indispensable for NIPS's performance, leading to a maximum decrease of R^2^ only by 0.02 (Figure 6B; see supplemental data file 2 for complete results). However, keeping only one area while removing all others greatly reduced NIPS's performance, causing a median drop of R^2^ by 0.29 even though the original R^2^ was only ≈0.24 (Figure 6C; see supplemental data file 3 for complete results). Taken together, these findings suggest that information about pain sensitivity is represented in a distributed neural system, rather than local and isolated regions.^[^ ^45^ ^]^

To further understand what NIPS represents, we then applied NIPS to predict pain reduction in healthy individuals using Datasets 3 and 6. Dataset 3 was originally collected to investigate placebo analgesia.^[^ ^39^ ^]^ We previously utilized the fMRI data in the control condition in Dataset 3 to test the generalizability of NIPS (Figure 5C). We now subtracted BOLD responses and mean pain ratings in the placebo condition from the control condition to quantify the placebo effect. NIPS significantly predicted the magnitude of placebo analgesia: 1) the 8 kg cm^−2^ condition: β = 0.205, *p* < 0.0001; 2) the 7 kg cm^−2^ condition: β = 0.166, *p* = 0.002; 3) the 6 kg cm^−2^ condition: β = 0.205, *p* < 0.0001 (Figure 6D). Dataset 6 was collected to study the analgesic effect of transcutaneous electrical nerve stimulation (TENS) with a pretest‐treatment‐posttest design.^[^ ^40^ ^]^ To quantify the analgesic effect, we subtracted BOLD responses and mean pain ratings in the posttest from those in the pretest. We also found a significant correlation between the model predictions and real pain reductions (r = 0.254, *p* = 0.015; Figure 6C). Furthermore, after accounting for the influence of baseline pain ratings (control condition in Dataset 3 and pretest condition in Dataset 5), we still found that predicted analgesic effects were significantly associated with real pain reductions: 1) the 8 kg cm^−2^ condition in Dataset 3: β = 0.156, *p* = 0.0005; 2) the 7 kg cm^−2^ condition in Dataset 3: β = 0.101, *p* = 0.017; 3) the 6 kg cm^−2^ condition in Dataset 3: β = 0.085, *p* = 0.040; 3) Dataset 6: partial r = 0.238, *p* = 0.023. Regressing dataset, sex, and age also had no substantial effect on the ability of the multivariate model to predict pain reduction in Datasets 3 and 6 (Figure S7D, Supporting Information). These findings converged to support the proposition that our fMRI‐based pain sensitivity model predicts, at least partly, pain reduction from interventions in healthy individuals.

### 2.5 NIPS has Comparable Performance as Established Pain Models

**Figure 7:**
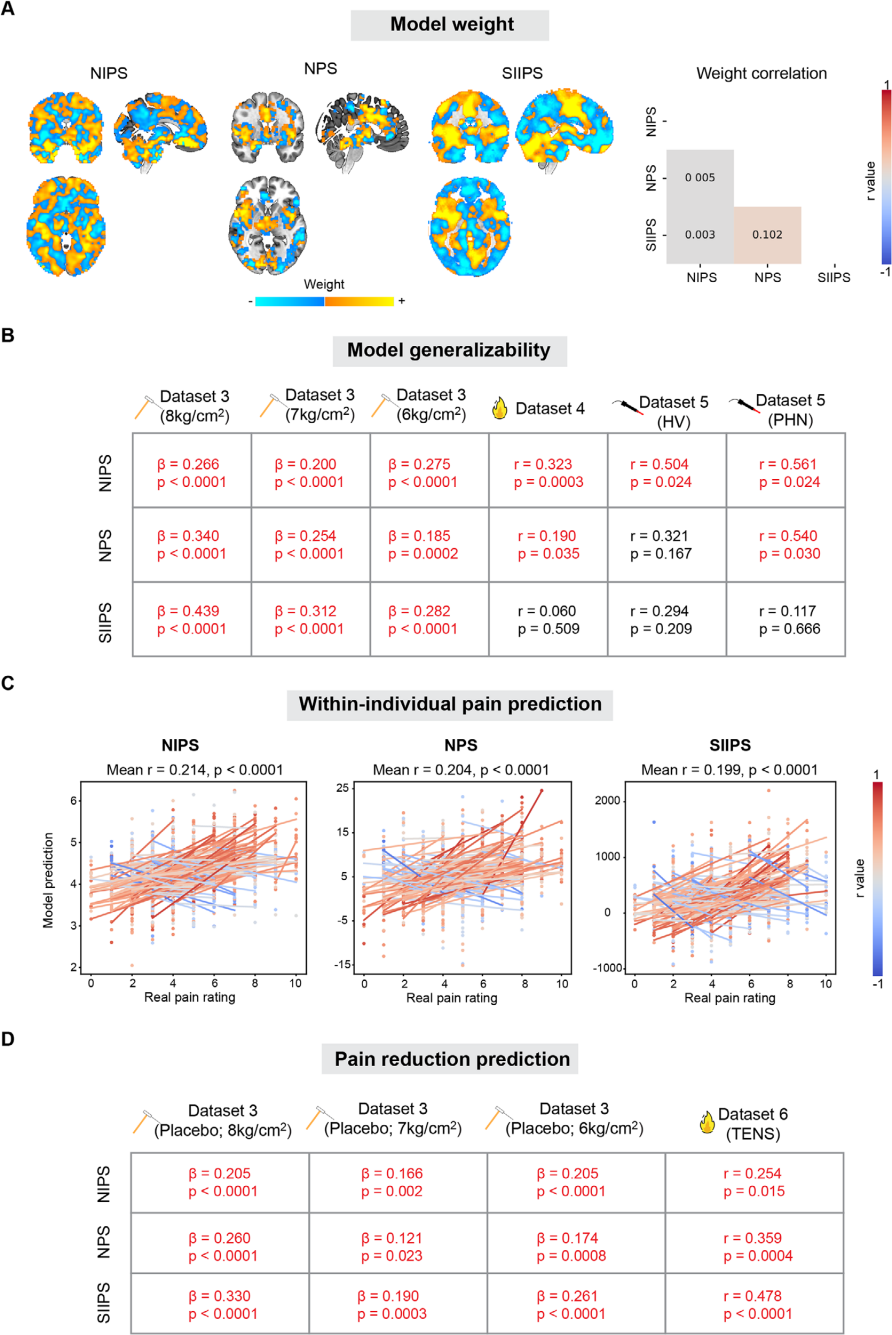
Comparisons between NIPS and established pain models. A) Weight maps of NIPS, NPS, and SIIPS. The three models have distinct weight maps. B) Model generalizability. NIPS has the best generalizability, whereas NPS and SIIPS failed to predict pain sensitivity in some datasets. C) Within‐individual pain rating prediction in the Holdout Test Set. All three models significantly predicted single‐trial pain ratings. D) Pain reduction prediction. All three models significantly predicted pain reduction induced by placebo and TENS. Significant prediction‐outcome correlations are marked in red in (B and D).

To contextualize our model, we compared it with well‐established pain prediction models, specifically the Neurological Pain Signature (NPS)^[^ ^46^ ^]^ and Stimulus Intensity Independent Pain Signature (SIIPS) (**Figure** **7**).^[^ ^47^ ^]^ Their weight maps had only negligible correlations, suggesting our model captures a distinct neural pattern and represents an independent model. We further applied NPS and SIIPS to our datasets to examine their predictive power relative to our model. Both NPS and SIIPS significantly predicted pain sensitivity in the Holdout Test Set (Figures S10 and S11, Supporting Information). However, they seemed less generalizable than NIPS, especially in Datasets 4 and 5, which included older participants and chronic pain patients (Figure 7C). Please note that NPS and SIIPS were developed to predict within‐individual pain states, whereas our model was trained to predict between‐individual pain sensitivity. To examine if these models could equally predict within‐individual and between‐individual pain, we then applied them to single‐trial data in the Holdout Test Set. All of them had some predictive power on single‐trial pain rating prediction. Like our model, NPS and SIIPS also predicted individual differences in pain reduction induced by placebo and TENS. These systematic comparisons suggest that one key strength of our model lies in its broad generalizability. In other aspects, it appears comparable to the well‐accepted NPS and SIIPS models.

## 3 Discussion

With six large and ethnically and culturally diverse fMRI datasets, we systematically examined the relationship between nociceptive‐evoked BOLD responses and pain sensitivity across individuals. We obtained four major findings. First, there were replicable and generalizable correlations between nociceptive‐evoked BOLD responses and pain sensitivity. Second, the correlations between BOLD responses and sensory sensitivity were more evident in pain than in other modalities. Third, NIPS accurately predicted not only pain sensitivity across different pain types, but also pain relief from different pain treatments. Fourth, sample sizes had enormous impacts on assessing the relationship between BOLD responses and pain sensitivity using both univariate correlation analysis and multivariate predictive modeling. Altogether, using the largest dataset on nociceptive‐evoked fMRI responses available to date, we demonstrate that BOLD responses can encode pain sensitivity across various types of pain. These findings would facilitate interpretations of subjective pain reports and promoting more mechanistically informed investigations into pain physiology.

Understanding the neural basis of variability of pain sensitivity has been a major topic in pain neuroscience.^[^ ^10^, ^11^, ^12^, ^13^, ^14^, ^27^, ^48^, ^49^, ^50^ ^]^ Theoretically, nociceptive‐evoked BOLD responses reflect real‐time neural processing of painful stimuli, thus potentially having a stronger correlation with pain sensitivity than other brain features, such as grey matter volume,^[^ ^48^ ^]^ cortical thickness,^[^ ^50^ ^]^ and resting‐state functional connectivity.^[^ ^12^, ^13^ ^]^ Indeed, task fMRI data do improve the prediction of cognitive traits compared with resting‐state fMRI data.^[^ ^51^, ^52^ ^]^ However, it is still controversial whether nociceptive‐evoked BOLD responses can encode pain sensitivity, since inconsistent findings were obtained in different groups even when the sample size (≈100) seemed large.^[^ ^11^, ^18^ ^]^ Leveraging six datasets with a total sample size of 1046, we found that nociceptive‐evoked BOLD responses correlated with pain sensitivity. This correlation was analytically robust with respect to statistical methods (parametric or nonparametric) and potential confounders (dataset heterogeneity, sex, and age), replicable in multiple datasets with different stimulus intensities, and generalizable across different pain types in ethnically and culturally diverse populations. In agreement with previous studies,^[^ ^10^, ^16^, ^18^ ^]^ typical pain‐related areas were also among the areas encoding pain sensitivity. We thus provided compelling evidence that, given sufficiently large sample sizes, pain sensitivity could be indexed from BOLD responses to pain, which is consistent in principle with previous studies showing the ability of nociceptive‐evoked electrophysiological responses to encode pain sensitivity.^[^ ^27^, ^49^ ^]^

We also developed NIPS to predict pain sensitivity from nociceptive‐evoked BOLD responses. With multiple independent datasets, we confirmed that NIPS has good predictive performance. Cross‐validation and testing in the Holdout Test Set demonstrated that NIPS explained ≈20% of the variance of pain sensitivity. It is noteworthy that individual difference models based on large samples generally have only modest performance.^[^ ^25^, ^52^, ^53^, ^54^ ^]^ For example, in the Adolescent Brain Cognitive Development dataset, n‐back task fMRI‐based models explained ≈10% variance of various cognitive traits like working memory.^[^ ^54^ ^]^ Importantly, the performance of our model is slightly better than the established NPS and SIIPS models,^[^ ^46^, ^47^ ^]^ possibly due to the fact that our model is specifically built to predict between‐individual pain sensitivity whereas NPS and SIIPS are mainly for predicting within‐individual pain ratings. When applied to predicting within‐individual pain states, our model, NPS, and SIIPS all had similar significant performance. At least two explanations exist for this finding. One is that momentary pain states and relatively stable pain traits have shared neural mechanisms. Indeed, between‐individual pain‐sensitivity encoding regions respond to within‐individual pain states as well.^[^ ^15^, ^16^ ^]^ Additionally, our operationalization of pain sensitivity may contribute to the observation that models built with distinct purposes predicted pain sensitivity and pain states. We employed average pain ratings across trials as a metric of pain sensitivity.^[^ ^1^ ^]^ Models predicting pain ratings may thus transfer part of its predictive power to pain sensitivity and vice versa. Given the linear nature of our model, it is unlikely to fully dissociate within‐individual (state‐related) and between‐individual (trait‐related) effects. However, given the better performance on between‐individual pain sensitivity, our model seems to primarily capture individual differences in pain sensitivity, rather than transient fluctuations in pain perception.

Crucially, we tested NIPS's generalizability by applying it to multiple external datasets. Unsurprisingly, its performance dropped, as machine learning models’ performance generally decreases in external datasets that differ greatly from the discovery set.^[^ ^55^ ^]^ Nevertheless, NIPS still exhibited significant predictive power in these datasets, which is particularly remarkable considering the amount of heterogeneity in our external datasets. Variability existed not only in the types of pain (mechanical pain in Dataset 3, contact heat pain in Dataset 4, and laser heat pain in Dataset 5), but also in the pain rating scale (0–1 Labeled Magnitude Scale in Datasets 3 and 4, 0–10 Numeric Rating Scale in Dataset 5), perceived pain intensity, MRI specifications, and fMRI sequences. Furthermore, participants differed in age (relatively young in Datasets 3 and 4, and older individuals with mean age>60 years in Dataset 5), health condition (healthy in Datasets 3 and 4, and chronic pain patients in Dataset 5), and cultural background (Chinese in Datasets 1, 2, 5; Americans in Dataset 3; and South Koreans in Dataset 4). Such dataset/site‐related variability may confound fMRI findings in multi‐site studies like ours.^[^ ^56^, ^57^, ^58^ ^]^ However, we did not pool all these datasets, but analyzed them separately except for Datasets 1 and 2. In other words, all analyses were conducted on a homogeneous dataset except for those involving Datasets 1 and 2. Since Datasets 1 and 2 were collected from the same site on a rolling basis and differed only in pain sensitivity and laser stimulation parameters, the site effect should be minimal. Consequently, dataset heterogeneity does not confound our results, but rather demonstrates that our findings can be generalized across various experimental settings and populations. Nonetheless, it warrants further investigation to determine whether, and to what extent, the site effect contributed to the performance drop of our model in external validation sets. Importantly, NIPS predicted pain sensitivity in PHN patients in Dataset 5, suggesting some potential clinical relevance of our model, although its performance in patients with chronic pain still requires further testing in more clinical datasets. This broad generalizability is a key strength of our model. Well‐established NPS and SIIPS models also predicted pain sensitivity in some datasets, but failed to do so in other datasets, especially in Dataset 5, which is more representative of the population with pain problems.

Given these generalizable findings, why did some studies fail to find any correlation between BOLD responses and pain sensitivity? We examined two likely explanations. One is that the physical intensity of painful stimuli might be too small to evoke a clear pain sensation.^[^ ^59^ ^]^ The statistical power to detect correlations will be dampened if no clear pain sensation is elicited or BOLD responses are just too noisy due to insufficient physical intensity. Our results from Dataset 3 supported this explanation: fewer areas showed significant correlations with pain sensitivity as the physical intensity decreased. The other explanation is that the sample size might be simply not large enough. Traditionally, neuroimaging studies have limited sample sizes,^[^ ^60^, ^61^ ^]^ which is a crucial factor in the non‐replicability of study findings.^[^ ^23^, ^25^ ^]^ Taking advantage of our large datasets, we systematically investigated the impact of sample size on the detectability of univariate correlations between nociceptive‐evoked BOLD signals and pain sensitivity with resampling analysis. We found that a sample size of around 200 could only detect less than 25% of the significant voxels in univariate whole brain correlation analysis with 80% statistical power. Due to the reduced number of statistical tests, a priori ROI analyses required smaller sample sizes, but still needed more than 100 in areas with large effects (e.g., cerebellum). Similarly, sample sizes also had a substantial influence on the predictive power of NIPS. Only when the sample size reached 150 did we observe stable predictive performance. Beyond this number, NIPS's predictive performance improved continuously as the sample size grew, suggesting that better models can be built with even more data.^[^ ^62^, ^63^, ^64^ ^]^

Our findings resonate with recent much‐discussed studies arguing that a massive sample size like a few thousands is required to detect replicable brain‐behavior associations.^[^ ^24^, ^25^ ^]^ These studies have been the center of many commentaries, attracting both supports and critiques.^[^ ^65^, ^66^, ^67^, ^68^, ^69^ ^]^ In our study, detecting univariate brain‐pain sensitivity associations needs sample sizes of around 200 and building generalizable multivariate models is feasible with a sample size of 150—both far less than thousands. The discrepancy may result from our task‐based fMRI study design and focus on pain sensitivity. Task‐based fMRI studies can have larger effect sizes than resting‐state fMRI studies,^[^ ^51^, ^52^ ^]^ which has been the main focus of previous fMRI‐behavior association studies claiming that thousands of participants are necessary.^[^ ^24^, ^25^ ^]^ Pain sensitivity also exhibits dramatic between‐individual variability and greater variability in behavioral measures reduces the sample size needed.^[^ ^70^ ^]^ Distinct sample size estimates notwithstanding, the general conclusion remains the same: sufficient sample sizes are important for replicability in individual difference neuroimaging studies.

Previous studies have shown that painful and nonpainful stimuli activate roughly the same brain regions,^[^ ^28^, ^30^, ^33^ ^]^ which was replicated in our study, suggesting that BOLD responses may not selectively reflect pain. However, most previous studies have mainly focused on the relationship between fMRI responses and pain perception at the within‐individual level.^[^ ^31^, ^32^, ^33^, ^34^ ^]^ Our study advances this line of research by investigating whether fMRI responses differentially encode pain sensitivity at the between‐individual level. We found significant correlations between nonpainful stimuli‐evoked BOLD responses and corresponding sensory sensitivity, which agrees with a recent study showing correlations between BOLD responses and auditory sensitivity.^[^ ^11^ ^]^ Further analyses showed larger correlations between BOLD responses and pain sensitivity than between BOLD signals and tactile/auditory/visual sensitivity. These findings suggest that, although fMRI signals seem more capable of indexing pain sensitivity, the associations between BOLD responses and sensory sensitivity are not unique to pain. One reason for this finding could be that painful and nonpainful stimuli share some common attributes. Identifying pain selective neural indicators is challenging unless the common factors can be removed beforehand. One of the common factors is general rating bias.^[^ ^71^ ^]^ Individuals who tend to report higher ratings will be classified as having higher pain sensitivity as well as higher nonpain sensitivity.^[^ ^72^ ^]^ As we have shown previously, control over rating bias with signal detection theory yields more pain‐selective findings.^[^ ^14^ ^]^ Another common factor is salience. All transient stimuli are believed to be salient,^[^ ^73^ ^]^ although painful stimuli may be inherently more salient and attention‐grabbing than nonpainful stimuli. Salience may partly explain the seemingly differential representations of pain sensitivity and nonpain sensitivity. Without salience data, we are in principle unable to directly test the role of stimulus salience on our findings. Future research collecting salience ratings or physiological signals is in need to directly test the contribution of stimulus salience.^[^ ^74^, ^75^ ^]^

Apart from demonstrating the ability of BOLD responses to pain sensitivity, the present study also deepens our understanding of neural representations of pain sensitivity. Univariate analyses revealed direct correlations between pain‐evoked fMRI responses and pain sensitivity, suggesting a magnitude coding scheme of pain sensitivity, that is, individuals more sensitive to pain have larger brain responses in pain‐related areas, such as the S1, S2, ACC, insula, thalamus, and cerebellum.^[^ ^10^ ^]^ The role of cerebellum is particularly intriguing. It has one of the largest correlation coefficients with pain sensitivity and requires a relatively small sample size to detect its associations with pain sensitivity compared to other pain‐related areas. Moreover, it is one of the most consistent pain‐activated areas and also contributes to established pain models, such as NPS and SIIPS.^[^ ^16^, ^46^, ^47^ ^]^ However, the cerebellum has historically received little attention and discussion in the pain literature.^[^ ^76^, ^77^ ^]^ Given its widespread connection with the cerebral cortex,^[^ ^78^ ^]^ future studies may further test whether and how cerebellar‐cerebral interactions encodes pain sensitivity. In addition to univariate results, multivariate modeling shows that voxels predictive of pain sensitivity were distributed all over the brain. When removing brain areas one by one, we observed few to no reductions in NIPS's predictive power. On the other hand, keeping only one area led to great reductions in the predictive performance of NIPS. These findings imply that, apart from magnitude coding, pain sensitivity seems to be population coded. Specifically, pain sensitivity is likely to be encoded in a largely distributed manner rather than in some localized brain areas,^[^ ^45^ ^]^ which is in agreement with real brain lesion studies showing that lesions in key pain‐related areas do not disrupt pain intensity perception.^[^ ^79^, ^80^ ^]^ Importantly, albeit with similar predictive performance, NIPS seems to be a complementary model orthogonal to NPS and SIIPS, suggesting that the brain may represent pain with multiple independent patterns. One reason for the independence of these models could be that they are trained with different data to predict different aspects of pain,^[^ ^46^, ^47^ ^]^ but further studies are needed to systematically examine the quality of their differences and the reasons and implications of multiple pain sensitivity representations in the brain.

One particularly interesting finding was that NIPS could predict individual differences in pain reduction from different interventions. Similarly, NPS and SIIPS also predicted individual differences in pain reduction, which is consistent with a recent study.^[^ ^39^ ^]^ These findings may have profound theoretical and practical implications. Theoretically, they bridge two critical lines of pain research: pain sensitivity and pain modulation. Successful cross prediction indicates that individual differences in pain sensitivity may be related to individual differences in analgesic effects, which is supported by previous studies showing significant associations between pain sensitivity and conditioned pain modulation.^[^ ^81^, ^82^ ^]^ Neurobiologically, the perception of pain reflects a balance between ascending nociceptive input and descending modulatory influences.^[^ ^83^ ^]^ Our findings thus could mean that pain sensitivity may reflect the influence of descending pain modulation on pain sensitivity for each individual. This statement is in line with the finding that NIPS, NPS, and SIIPS all explained more variance of pain sensitivity than the variance of analgesic effects in Dataset 3. Alternatively, NIPS's ability to predict pain reduction could also reflect the overlap in brain regions involved in both pain modulation and pain processing, e.g., the rACC.^[^ ^84^ ^]^ Future studies can investigate deeper relationships between pain sensitivity and individual differences in analgesic effects. Practically, our findings may suggest a potential application of pain models: identifying patients who respond positively to pain treatments, such as placebo interventions.^[^ ^5^ ^]^ This potential application is particularly important given the scarcity of models predicting pain reduction relative to those predicting pain sensitivity.^[^ ^85^ ^]^ If pain sensitivity models can be transferred to predicting analgesic outcomes, personalized pain treatment strategies could benefit directly from existing pain sensitivity model. To achieve this practical goal, future studies should further optimize the performance of NIPS and related models and rigorously evaluate their predictive capabilities for individual differences in analgesic effects in clinical settings.

Our study has some limitations. First, we only investigated the influence of stimulus intensity and sample size on the detectability of the correlation between BOLD responses and pain sensitivity due to the nature of available datasets. Other contributing factors like sample personal characteristics and study design need to be tested in future studies. Second, while NIPS has reasonably good performance in predicting pain sensitivity, its predictive power can be further improved. As suggested by our resampling analysis, collecting even more data has the potential to boost NIPS's predictive performance.^[^ ^86^ ^]^ The improved performance of our composite model integrating fMRI and behavioral measures also suggest that incorporating multimodal data may be helpful to capture more variability of pain sensitivity.^[^ ^64^ ^]^ Since fMRI alone may not be sufficient to explain the majority of pain sensitivity, data from other imaging modalities are most likely indispensable to build a model applicable in real life. Finally, the clinical implications of our study are limited, since participants in our study were mainly healthy individuals. Clinical populations are typically more variable in terms of age, socioeconomic background, education, diagnosis, and symptoms. While our datasets, especially the PHN dataset (i.e., Dataset 5), exhibit some variability in age, ethnicity, cultural background, and pain diagnosis, our findings are mainly based on homogeneous samples, and thus still need to be replicated in more heterogeneous clinical populations. Although we demonstrated NIPS's ability to predict pain sensitivity in PHN patients, it remains to be tested more extensively in other chronic pain patients, and it is of particular interest to test whether NIPS can predict pain relief in patient groups.

## 4 Conclusion

With the largest nociceptive‐evoked fMRI datasets to date, we revealed that, given sufficient sample sizes, BOLD responses can encode pain sensitivity across various types of pain better than non‐pain sensitivity. Furthermore, we built a highly replicable and generalizable fMRI‐based indicator of pain sensitivity across individuals.

## 5 Experimental Section

### Datasets Overview

Six fMRI datasets were utilized in the present study (Figure 1A). Datasets 1, 2, 5, and 6 were collected using E‐Prime (ver. 2.0, PST Inc.) in China. Datasets 3 and 4 were collected using Matlab (ver. 2018b) in the US and South Korea, respectively, and kindly shared by the original authors.^[^ ^18^, ^39^ ^]^ In Datasets 1 and 2, participants received laser pain, electro‐tactile, auditory, and visual stimuli.^[^ ^14^ ^]^ Dataset 3 was originally collected to investigate the neural underpinnings of placebo analgesia.^[^ ^39^ ^]^ The researchers delivered both contact heat pain and mechanical pain stimuli to participants, but used contact heat pain stimuli to induce placebo analgesia by conditioning. To avoid the potential influence of the placebo induction on the neural processing of contact heat pain, data were analyzed only for the mechanical pain stimuli in the placebo and control conditions, and discarded data with contact heat pain stimuli. Instead, data were used with contact heat pain stimuli from Dataset 4,^[^ ^18^ ^]^ where no additional interventions like placebo were introduced. Dataset 5 delivered laser heat pain stimuli to postherpetic neuralgia (PHN) patients and healthy controls.^[^ ^38^ ^]^ Dataset 6 also used contact heat pain stimuli, and was from a study aiming to reveal the spino‐cortical mechanisms of TENS‐induced analgesia.^[^ ^40^ ^]^ In Datasets 1–5, the physical intensity of painful stimuli was fixed. In Dataset 6, however, the physical intensity was adjusted individually for each participant. Therefore, Dataset 6 was not included in most analyses, but only applied NIPS to it to test whether NIPS could predict pain reduction. Basic information was summarized for these six datasets in Table S1 (Supporting Information).

### Participants

Dataset 1 included 212 participants. One participant did not provide the necessary demographic information. For the rest 211 participants, there were 135 females, aged 21.5 ± 4.2 years (Mean ± SD). Dataset 2 had 187 participants. There was also one participant who did not provide the demographic information. For the rest 186 participants, there were 103 females, aged 21.0 ± 3.3 years. Note that Datasets 1 and 2 were collected simultaneously, not separately, in a single project. Treating them as two datasets reflects the fact that participants in these datasets had different pain sensitivity as assessed in a calibration phase and received painful stimuli of different intensities to avoid delivering pain stimuli that could be potentially intolerable for participants with high pain sensitivity. In other words, participants were recruited on a rolling basis and assigned to either Dataset 1 or Dataset 2 based on their pain sensitivity, which was assessed during the calibration phase. Dataset 3 included 395 participants (231 females, age: 35.4 ± 2.6). Note that some participants in this dataset had missing behavioral ratings in some conditions. Considering the lack of sufficient trials per condition, data were discarded from these participants with missing values, which was the reason why the sample sizes in the main figures were 369, 358, and 371 for the three intensity conditions respectively. Dataset 4 had 124 participants (61 females, age: 22.2 ± 2.7). Dataset 5 included 16 right‐handed patients (5 males; age [mean ± SD] = 65.75 ± 6.99 years) suffering from one condition of chronic pain called postherpetic neuralgia (PHN) and 20 age‐ and gender‐matched right‐handed healthy controls (8 males; age: 61.55 ± 8.21 years). Dataset 6 included 92 participants (50 females, age: 21.9 ± 3.2). All participants except for Dataset 5 were healthy and free of pain, neurological, or psychiatric disorders. Patients in Dataset 5 fulfilled the International Association for the Study of Pain (IASP) criteria for PHN and were diagnosed by experienced clinicians based on clinical symptoms (including medical history, shingles history, pain severity, and pain types). None of participants in Dataset 5 had a past or current diagnosis of any psychiatric or major neurological illness. All participants gave written informed consent prior to the experiments. Local ethics committees approved the experimental procedures for the original studies (Datasets 1, 2, 5, and 6: Ethics Committee of the Institute of Psychology at the Chinese Academy of Science; Dataset 3: Institutional Review Board of the University of Colorado Boulder; Dataset 4: Sungkyunkwan University Institutional Review Board).

### Sensory Stimulation

Three different types of painful stimuli were used in these datasets: 1) laser heat pain: Datasets 1, 2, and 5; 2) mechanical pain: Dataset 3; 3) contact heat pain: Datasets 4 and 6. Datasets 1 and 2 also delivered three kinds of nonpainful stimuli: 1) electro‐tactile, 2) auditory, and 3) visual stimuli.

In Datasets 1 and 2, laser heat pain stimuli were transient radiant heat pulses (wavelength: 1.34 µm; pulse duration: 4 ms) generated by an infrared neodymium yttrium aluminum perovskite (Nd: YAP) laser (Electronical Engineering, Italy). The laser beam was transmitted by an optic fiber, and its diameter was set at ≈7mm. Laser pulses were delivered to a pre‐defined square (5 × 5cm^2^) on the left‐hand dorsum. After each stimulus, the laser beam was displaced by ≈1cm in a random direction to avoid nociceptor fatigue or sensitization. Two stimulus energies (3.0 and 3.5J) were used in Dataset 1, and two other stimulus energies (3.5 and 4.0J) were used in Dataset 2. A stimulus intensity of 3.0J was adopted in Dataset 1 because participants in this dataset had higher pain sensitivity and nociceptive laser stimuli of 4.0J could be unbearable for them. In Dataset 3, mechanical pain stimuli were delivered to finger nails of the left hand for 10s with an MRI‐compatible pressure pain device. There were three levels of physical intensity, 6, 7, and 8 kg cm^−2^. In Datasets 4, contact heat pain stimuli were administered to the volar surface of the left forearm with a 16 × 16mm^2^ ATS thermode of a Pathway system (Medoc Ltd, Ramat Yishai, Israel). The target temperature of the stimuli was 47.5 °C with the baseline temperature being 32 °C. The stimulation lasted for 12s (ramp‐up: 2.5s; plateau: 7s; ramp‐down: 2.5s). In Dataset 5, laser heat pain stimuli were also transient radiant heat pulses. Stimulus parameters were similar to those in Datasets 1 and 2, except that the laser energy was only set to 3.5J. In Dataset 6, contact heat pain stimuli were delivered to the C5–C6 dermatomes on both forearms with a 573mm2 (diameter of 27mm) CHEPS thermode using a Pathway system (Medoc Ltd, Ramat Yishai, Israel). The heat stimulation ramped up from a baseline of 32 °C to a temperature for 3s that led to a perceived intensity rating of 7 on a 0–10 rating scale. In other words, the target temperature was tailored individually for each participant to ensure a constant perceived intensity. Laser heat and contact heat stimuli both evoke heat pain, but differ in two main aspects. First, laser stimuli can selectively activate nociceptors without eliciting tactile sensations while contact heat stimuli inevitably activate the touch receptors.^[^ ^87^ ^]^ Consequently, brain activities evoked by contact heat could be less specific to nociception compared with those evoked laser heat. Second, laser stimuli used in Datasets 1 and 2 were extremely brief (4ms), while contact heat stimuli in Datasets 4 and 6 lasted for several seconds (12s in Dataset 4 and 3s in Dataset 6). Longer‐duration nociceptive stimuli generally evoke stronger emotional unpleasantness, which is related to areas like the (anterior) insula and ACC.^[^ ^88^ ^]^

In Datasets 1 and 2, nonpainful stimuli were also delivered. Non‐nociceptive tactile stimuli were constant current square‐wave electrical pulses (duration: 1ms; model DS7A, Digitimer, UK) delivered through a pair of skin electrodes (1 cm interelectrode distance) placed on the left wrist, over the superficial radial nerve. The same two stimulus intensities (2.0 and 4.0 mA) were used in both datasets. Auditory stimuli were brief pure tones (frequency: 800 Hz; duration: 50 ms; 5 ms rise and fall time) delivered through a headphone. The same two stimulus intensities (76 and 88 dB SPL) were used for all participants in both datasets. Visual stimuli were brief flashes of a grey round disk in a black background (duration: 100ms) on a computer screen. The stimulus intensities were adjusted using the greyscale of the round disk, corresponding to RGB values of (100, 100, 100) and (200, 200, 200), respectively, for all participants in both datasets. Stimulus intensities of tactile, auditory, and visual stimuli were determined based on a pilot behavioral experiment to ensure that the perceived ratings of low and high intensity stimuli were ≈4 and 7 out of 10, respectively.

Note that since the physical properties of stimuli were held constant for Datasets 1–5, this study design ensures behavioral and neural responses in the analyses would reflect individual differences were conducted rather than within‐individual level differences.

### Rating Scale

Two ratings scales were used for different datasets: 0–10 Numeric Rating Scale and 0–1 Labeled Magnitude Scale. In Datasets 1, 2, 5, and 6, the 0–10 Numeric Rating Scale was used, where 0 meant “‘no sensation”’, and 10 “‘the strongest sensation imaginable”’. This is a common rating scale in pain research, but evidence has suggested that it is not a ratio scale, namely, a rating of 4 might not be exactly twice as painful as a rating of 2.^[^ ^89^ ^]^ In Datasets 3 and 4, the 0–1 Labeled Magnitude Scale was adopted. In Dataset 3, 0 on the scale meant “no pain”, 0.014 “barely detectable”, 0.061 “weak pain”, 0.172 “moderate pain”, 0.354 “strong pain”, and 1 “most intense sensation imaginable”.^[^ ^39^ ^]^ In Dataset 4, 0 meant “no sensation”, 0.061 “weak”, 0.172 “moderate”, 0.3454 “strong”, 0.533 “very strong”, and 1 “strongest imaginable”.^[^ ^18^ ^]^ The Labeled Magnitude Scale was not used as often as the Numeric Rating Scale in pain research, but seems to be a ratio scale.^[^ ^90^ ^]^ All participants were asked to rate the intensity of pain according their authentic sensory experience without any other considerations. Note that preexisting datasets were analyzed. As a result, this study had no control over which rating scale to use. Additionally, due to differences in the perceptual meaning of ratings, the 0–1 scale could not be multiplied by 10 to convert it to a 0–10 scale.

### Study Design

The original authors reported their study designs in great detail.^[^ ^14^, ^18^, ^38^, ^39^, ^40^ ^]^ Since these papers were all openly accessible, here the study design of each dataset (Figure 1B) was only briefly described.

In Datasets 1 and 2, participants first underwent a quantitative sensory testing and filled out a series of questionnaires (see Zhang et al.^[^ ^91^ ^]^ for details). The quantitative sensory testing assessed laser heat pain threshold, and cold pain threshold and tolerance. Questionnaires included the Chinese versions of Fear of Pain Questionnaire (FPQ), Pain Anxiety Symptoms Scale‐20 (PASS), Pain Catastrophizing Scale (PCS), Pain Vigilance and Awareness Questionnaire (PAVQ), Beck Depression Inventory (BDI), Behavioral Activation and Behavioral Inhibition Scales (BAS/BIS), Revised Life Orientation Test (LOT‐R), Pittsburgh Sleep Quality Index (PSQI), Interpersonal Reactivity Index (IRIC), Big Five Personality Test, and State‐Trait Anxiety Inventory (STAI). Then participants received 80 transient stimuli of four different sensory modalities (nociceptive laser, non‐nociceptive tactile, auditory, and visual) divided into two runs, and then rated their perceived intensity with a rating scale ranging from 0 (“‘no sensation”’) to 10 (“‘the strongest sensation imaginable [in each stimulus modality]”’). For each sensory modality, two stimulus intensities (i.e., high and low) were delivered. In other words, each participant underwent eight experimental conditions (4 modalities×2 intensities), each condition with 10 trials. The only difference in the study design of Datasets 1 and 2 was the physical intensity of laser heat pain stimuli (detailed in Sensory stimulation).

Dataset 3 adopted a within‐subjects design to examine the neural mechanisms of placebo analgesia. The study consisted of a verbal suggestion phase, a conditioning phase, and a test phase. Verbal suggestions and conditioning were used to induce placebo effects. In the verbal suggestion phase, two identical creams were introduced to participants, one being a placebo cream that was claimed to have a pain‐relieving effect, the other being a control cream claimed to have no analgesic effects. These two creams were applied to two different fingers of the left hand. Then participants went through the conditioning phase, where they experienced the analgesic effect of the placebo cream through social observational learning and classical conditioning. Importantly, in this conditioning phase, only contact heat pain stimuli were delivered. Finally, in the test phase, participants received both contact heat pain and mechanical pain stimuli in the fingers with the placebo and control creams. The test phase delivered a total of 32 stimuli in four runs, with four thermal and four mechanical stimuli in a random order per run. After each stimulus, participants rated the intensity of the stimulus with a 0–1 Labeled Magnitude Scale, a quasi‐logarithmically spaced perceptual verbal labels rating scale, where 0 means “no pain”, 0.014 “barely detectable”, 0.061 “weak pain”, 0.172 “moderate pain”, 0.354 “strong pain”, and 1 “most intense sensation imaginable”.

In Dataset 4, participants received contact heat pain stimuli of six intensity levels in their left forearm. The entire study consisted of eight pain task runs with 12 trials each run and 16 trials per temperature. After each stimulus, participants rated the perceived intensity of the stimuli with a 0–1 Labeled Magnitude Scale. Note that the original authors only shared data for the highest temperature condition (i.e., 47.5 °C). Thus, data could only be analyzed for this condition.

In Dataset 5, participants received 20 laser heat pain stimuli and reported their perceived intensity and unpleasantness ratings with a 0–10 Visual Analog Scale (VAS), with 0 indicating “no sensation” and 10 indicating “unbearable sensation”. Only intensity ratings were analyzed to focus on the sensory aspect of pain.

Different from Datasets 1–5, Dataset 6 adopted a between‐subjects design to investigate the cortico‐spinal mechanisms of TENS‐induced analgesia. Participants were randomly assigned to three groups: conventional TENS (c‐TENS), acupuncture‐like TENS (a‐TENS), and sham TENS. Each participant experienced 60 contact heat pain stimuli (pre‐treatment session: 15 stimuli in the left arm and 15 stimuli in the right arm; post‐treatment session: 15 stimuli in the left arm and 15 stimuli in the right arm) in two runs and then reported their perceived intensity and unpleasantness in a 0–10 rating scale (0: “no feeling/unpleasantness”; 10: “unbearable pain/extreme unpleasantness”). Note that only data were used from the 15 stimuli delivered to the left arm, since painful stimuli were only delivered to the left hand in other datasets. Data were used from all three groups. The purpose was twofold. First, a large sample size could be had and thus a larger statistical power. Second, goal was to test whether NIPS could predict the amount of pain reduction after the treatment compared with the pre‐treatment baseline, not the analgesic effect of verum TENS compared with sham TENS. Indeed, pain sensitivity could also change after the sham TENS treatment due to factors like natural history, regression to the mean, and so on. A model that predicts pain reduction should thus also predict sensitivity change in the sham TENS condition.

### MRI Acquisition

Details of the MRI acquisition parameters can be found in Tables S2 and S3 (Supporting Information).

### Image Preprocessing

Preprocessed data were analyzed from the original studies.^[^ ^14^, ^18^, ^38^, ^39^, ^40^ ^]^ Detailed preprocessing descriptions could be found in these papers. For Datasets 1 and 2, fMRI data were preprocessed using Statistical Parametric Mapping 12 (SPM12) (Wellcome Trust Center for Neuroimaging, London). The preprocessing included: 1) removing the first three volumes in each run; 2) slice‐time correction using the second slice and realignment to the mean slice; 3) co‐registration; 4) normalizing to the Montreal Neurological Institute (MNI) space (resampling voxel size = 3 × 3 × 3mm^3^); 5) regressing out five principal components of the white matter (WM) and cerebrospinal fluid (CSF) signals, and the six motion parameters; 6) smoothing with a 6 mm full‐width at half maximum (FWHM) Gaussian kernel.

For Dataset 3, images were preprocessed with fMRIprep ver. 20.2.3.^[^ ^92^ ^]^ Briefly, the preprocessing included: 1) motion‐correction with MCFLIRT (FSL 5.0.9); 2) co‐registering to the T1 reference using the boundary‐based registration method (BBR) (FreeSurfer); 3) normalizing to the standard space; 4) smoothing with a 6 mm FWHM Gaussian kernel.

For Dataset 4, preprocessing was performed with SPM12 and FSL, including 1) removing the first 18 volumes for each run; 2) realignment; 3) co‐registration; 4) normalizing to the MNI space (2 × 2 × 2 mm^3^); 5) smoothing with a 5 mm FWHM Gaussian kernel; 6) reducing motion‐related artifacts with the Independent Component Analysis‐based strategy for Automatic Removal Of Motion Artifacts; 7) removing data where the mean frame displacement (FD) of a run > 0.2 mm, or the FD of any volume >5 mm.

For Dataset 5, preprocessing was conducted with FSL, including 1) motion correction using MCFLIRT; 2) removal of non‐brain structures using Brain Extraction Tool; 3) spatial smoothing using a Gaussian kernel with a 5 mm FWHM, and high‐pass temporal filtering (cut off: 100 s); 4) Independent component analysis (ICA)‐based denoising was performed for each individual fMRI data to remove the artifacts, including head motion, white matter and cerebrospinal fluid noise, high‐frequency noise, slice dropouts, gradient instability, EPI ghosting, and field inhomogeneities.

For Dataset 6, preprocessing was also conducted using FSL, with the following steps: 1) physiological noise (i.e., respiratory and cardiac noise) regression with the physiological noise model toolbox; 2) distortion correction with the TOPUP tool; 3) slice timing correction with the *slicetimer* function; 4) motion‐correction with MCFLIRT; 5) brain extraction with the BET; 6) smoothing with a 5 mm FWHM Gaussian kernel; 7) high‐pass filtering (cutoff: 100s); 8) co‐registration with the BBR; 9) normalizing to the MNI space (2 × 2 × 2 mm^3^) with FNIRT.

### fMRI Data Analysis—First Level Analysis

First level contrast images were reanalyzed from the original studies.^[^ ^14^, ^18^, ^38^, ^39^, ^40^ ^]^ In Datasets 1 and 2, regressors in the first level analysis included eight conditions (4 modalities × 2 intensities) convolved with the canonical hemodynamic response function, its temporal derivatives, and six head motion estimates. Moreover, images were high‐pass filtered with a cutoff period of 128 s and accounted for temporal autocorrelations using the first‐order autoregressive model (AR(1)). In Dataset 3, regressors in the first level analysis included all experimental conditions convolved with the canonical hemodynamic response function, 24 motion parameters, and mean CSF signal. A high‐pass filter of 180s was also applied. In Dataset 4, a single trial approach was utilized. Regressors included every single pain trial convolved with the canonical hemodynamic response function, five principal components of WM and CSF signal, and a linear trend. Signals were also filtered with an 180 s high‐pass filter. Trials with a variance inflation factor > 3 were subsequently removed. Single trial beta maps were finally averaged to derive the beta map for painful stimulation. In Dataset 5, regressors included the onset of laser stimuli and rating period convolved with a gamma hemodynamic response function and their temporal derivatives. In Dataset 6, regressors included painful stimulation and rating period convolved with the canonical hemodynamic response function and its temporal derivatives.

### fMRI Data Analysis—Second Level Analysis

Pain sensitivity was measured with the average pain rating of stimuli of constant physical intensity, which is a common measure^[^ ^1^ ^]^ and has been employed in previous studies.^[^ ^10^, ^11^, ^93^, ^94^ ^]^ Since the physical intensity was fixed, the reported rating differences primarily reflect between‐individual rather than within‐individual differences. In Datasets 1 and 2, to examine the relationship between nociceptive‐evoked BOLD responses and pain sensitivity, first level beta maps were correlated in each pain condition with average pain ratings. The significance threshold was set at p(FDR) = 0.05 (two‐tailed). To test the analytical robustness of results, both Pearson's r and Spearman's rho were computed in the correlational analyses. In addition to whole brain analysis, an ROI analysis was also conducted. Specifically, mean signals were extracted from classical pain‐related areas,^[^ ^16^, ^17^ ^]^ namely, the S1, S2, ACC, insula, thalamus, and cerebellum using anatomically defined masks. The cerebellum mask was from Diedrichsen et al.,^[^ ^95^ ^]^ and other masks were derived from defined with the Harvard‐Oxford Atlas distributed with FSL.^[^ ^96^ ^]^ Note that the Harvard‐Oxford Atlas is a probabilistic atlas and probability maps were thresholded for each region at 50%.^[^ ^97^ ^]^

Although Datasets 1 and 2 were assumed to differ only in the pain sensitivity and laser stimulation parameters, there might still be some unintended systematic differences between them. To control the potential influence of these dataset differences, sex, and age, a revised partial correlation analysis was conducted while controlling for dataset, sex, and age. Specifically, first BOLD contrast estimates were standardized voxel‐wise and behavioral measures for each dataset separately, then regressed out dataset identity (dummy coded as 0 and 1 for Datasets 1 and 2), sex (dummy coded as 0 and 1 for males and females), and age from both BOLD contrast estimates and mean pain ratings, and finally correlated the residuals of BOLD contrast estimates and mean pain ratings. With this dataset‐wise standardization procedure, not only the difference in the mean of ratings and BOLD responses but also the difference in the range of these variables were accounted for. Note that this revised partial correlation analysis is equivalent to standardize variables dataset‐wise and run multiple regression with the standardized variables. Datasets 1 and 2 had two missing values in sex and age, which were imputed with the median value.

To test the pain selectivity of the correlation between BOLD responses and sensory sensitivity, the same correlational analysis was also performed for touch, audition, and vision in Datasets 1 and 2. To assess the overlapping between areas encoding pain and nonpain sensitivity, conjunction analysis was conducted and computed the Dice coefficient between thresholded correlation maps. The Dice coefficient between two binary images (A and B) is defined as:

(1) $$Dice\left(A,B\right)=\frac{2*\left|A\cap B\right|}{A+B}$$

Furthermore, the correlation coefficients between modalities were compared to directly test the significance of correlation differences with Steiger's z tests.^[^ ^98^ ^]^ If correlations between *j* and *k*, and between *h* and *m* in *n* subjects are compared, then the *z* statistic of the correlation difference is defined as:

(2) $$z=\frac{Z_{jk}-Z_{hm}}{\sqrt{\frac{2\left(1-c\right)}{n-3}}}$$

where

(3) $$Z_{jk}=\frac{1}{2}ln\left(\frac{1+r_{jk}}{1-r_{jk}}\right)$$

(4) $$Z_{hm}=\frac{1}{2}ln\left(\frac{1+r_{hm}}{1-r_{hm}}\right)$$

(5) $$c=\frac{\frac{1}{2}{\bar{r}}^{2}\left(r_{jh}^{2}+r_{jm}^{2}+r_{kh}^{2}+r_{km}^{2}\right)+r_{jh}r_{km}+r_{jm}r_{kh}-\bar{r}\left(r_{jh}r_{jm}+r_{kh}r_{km}+r_{jh}r_{kh}+r_{jm}r_{km}\right)}{{\left(1-{\bar{r}}^{2}\right)}^{2}}$$

(6) $$\bar{r}=\frac{r_{jk}+r_{hm}}{2}$$

Some participants in Dataset 3 were twins from the same family. To account for this observation dependence introduced by the family factor, mixed effects models were ran with random intercepts of family. Specifically, the following model (in Wilkinson notation) was adopted: *Pain ∼ BOLD + (1|family_ID)*. To control for the potential influence of sex and age, the following additional mixed effects model was also run: *Pain ∼ BOLD + sex + age (1|family_ID)*. The significance threshold was set at p(FDR) = 0.05 (two‐tailed). Overlapping between pain sensitivity encoding maps in Datasets 1 and 2 and Dataset 3 was also assessed with the Dice coefficient. As in Datasets 1 and 2, an ROI analysis of the S1, S2, ACC, insula, and thalamus was also conducted using anatomically defined masks defined with the Harvard‐Oxford Atlas distributed with FSL.

All statistical tests in the article were two‐sided. All second‐level analyses were conducted with nilearn (ver. 0.11.1; https://nilearn.github.io/stable/index.html).

### Resampling Procedure

A resampling method was used to examine the influence of sample sizes on the probability of detecting significant correlations between BOLD responses and pain sensitivity. In the pooled 3.5J data from Datasets 1 and 2 (N = 399), bootstrapped subsamples were generated from the whole dataset, that is, randomly selected data samples with replacement. The sample size of these subsamples ranged from 100 to 400 (in steps of 10), and, for each sample size, the resampling procedure was repeated 100 times. In each subsample, BOLD responses were correlated with pain sensitivity and thresholded the correlation maps with p(FDR) = 0.05. Since 100 subsamples were generated for each sample size, the probability of a voxel being significant under a certain sample size could be approximated as the number of times that voxel reached significance. Then the sample size needed was calculated for each voxel to pass the p(FDR) = 0.05 threshold with a probability of 0.8.^[^ ^41^ ^]^

To supplement this whole brain analysis, the resampling was also conducted in an ROI analysis manner. Specifically, anatomical masks were defined for the S1, S2, ACC, insula, thalamus, and cerebellum. Subsequently, 1000 bootstrapped subsamples were generated with sample sizes from 10 to 400 (in steps of 10). In this ROI analysis, the significance was set to α = 0.01 or α = 0.001, and then made line charts to fully show the relationship between sample sizes and the probability of significance for each ROI.

### Machine Learning—Model Development

The pooled 3.5J data were split randomly in Datasets 1 and 2 into a Discovery Set (N = 199) and a Holdout Test Set (N = 200). A LASSO‐PCR model was trained to predict pain sensitivity using first level t maps in the Discovery Set with Scikit‐learn (ver 1.3.0; https://scikit‐learn.org/stable/index.html). The LASSO is a regularization method that shrinks the linear regression coefficient estimates toward zero.^[^ ^99^ ^]^ Mathematically, LASSO regression minimizes the following loss function:

(7) $$Loss=\sum_{i=1}^{n}{\left(y_{i}-\beta_{0}-\sum_{j=1}^{p}\beta_{j}x_{ij}\right)}^{2}+\lambda \sum_{j=1}^{p}\left|\beta_{j}\right|$$

where *y_i_* is the predicted variable of participant *i*, β_0_ and β_*j*_ are the linear regression coefficient estimates, *x_ij_* is the *j*‐th predictor for participant *i*, λ is the tuning parameter, *n* is the number of participants, and *p* is the number of predictors.

T maps were first standardized across participants and then submitted to principal component analysis to reduce the feature dimensions. All principal components were retained and used them as features in the LASSO regression model (Figure 6A). Nested five‐fold cross‐validation was used to evaluate model performance in the Discovery Set. The predictive performance of the final model was assessed using two metrics: 1) Pearson's correlation coefficient between the predicted and real pain sensitivity, and 2) R^2^, a metric representing the proportion of variance explained by the model. R^2^ is defined as:

(8) $$R^{2}=1-\frac{\sum_{i=1}^{n}{\left(y_{i}-{\hat{y}}_{i}\right)}^{2}}{\sum_{i=1}^{n}{\left(y_{i}-{\bar{y}}_{i}\right)}^{2}}$$

where *y_i_* is the real value for *i*‐th data point, ${\hat{y}}_{i}$ is the predicted value, and ${\bar{y}}_{i}$ is the mean of all real values. Note that R^2^ is not identical to the square of Pearson's correlation, and can be negative if model performance is worse than a simple mean prediction, that is, using ${\bar{y}}_{i}$ as predictions for all data points.

To determine the optimal tuning parameter λ, 100 λ values were selected log‐uniformly distributed in the [10^−2^, 102] interval and tuned λ according to the cross‐validated mean squared errors. The optimal λ was 0.236.

To examine the influence of dataset heterogeneity in the pooled 3.5J data in Datasets 1 and 2, dataset, sex, and age effects were regressed out from first level t maps and mean pain ratings (i.e., pain sensitivity), and built a LASSO‐PCR model. Note that to avoid data leakage issues, t maps and mean pain ratings were not standardized within dataset separately, but only the effect of dataset (dummy coded as 0 and 1 for Datasets 1 and 2), sex, and age was regressed out. Nested five‐fold cross‐validation was also used to evaluate model performance and tune the parameter λ from 100 values log‐uniformly distributed in [10^−2^, 102]. To determine which variable contributed to the dataset heterogeneity, models were also fitted controlling for only dataset, sex, or age in a similar manner.

### Machine Learning—Model Testing

After NIPS was developed, its performance was tested in the Holdout Test Set. To compare NIPS and other well‐recognized models, the Neurological Pain Signature (NPS)^[^ ^46^ ^]^ and Stimulus Intensity Independent Pain Signature (SIIPS)^[^ ^47^ ^]^ was applied to the Holdout Test Set. The predicted values (model expression) of these three models were the dot product of the weight maps of the models and the first‐level t map. NIPS was developed with laser heat pain data in Datasets 1 and 2. To test its generalizability, the model was then applied to mechanical pain data in Dataset 3 and contact heat pain data in Dataset 4. Note that the original authors of Datasets 3 and 4 shared beta maps for pain, not t maps. NIPS's performance in these two datasets may thus be slightly affected due to scaling issues in beta maps. To test NIPS's ability to predict pain sensitivity in chronic pain patients, it was applied to Dataset 5, which including PHN patients.

To examine the influence of sample size on NIPS's performance, the model with different training sample sizes was refitted with the “*learning_curve*” function in Scikit‐learn. The *learning_curve* function generates a curve showing how the performance of the model changes as the training sample size grows. First the pooled 3.5J data were randomly shuffled in Datasets 1 and 2, and then inputted the model and the shuffled dataset to the *learning_curve* function. Training sample sizes from 20 to 300 (in steps of 5) were considered. This whole process was repeated 100 times to also estimate the variability of the relationship between training sample size and model performance.

### Machine Learning—Model Interpretation

To understand each brain area's contribution to NIPS, the virtual lesion approach was employed.^[^ ^100^ ^]^ Specifically, NIPS's weight map was parcellated according to the Automatic Anatomical Labeling (AAL) template^[^ ^44^ ^]^ and removed areas one by one or kept areas one by one to examine each area's contribution to the model performance. To assess the potential association of NIPS with pain modulation, NIPS was also applied to Datasets 3 and 6 to predict their analgesic effects from different pain treatments in healthy individuals. To address observation dependence in Dataset 3, the following model was adopted to evaluate the relationship between real and predicted pain reduction: *real_value ∼ predicted_value + (1|family_ID)*. To control for the potential influence of sex and age, the following additional mixed effects model was also run: *real_value ∼ predicted_value + sex + age (1|family_ID)*.

### Machine Learning—Ensemble Model Combining fMRI and Behavior Measures

To assess whether fMRI data together with behavioral data contribute to a composite model that better explains variance of pain sensitivity, an ensemble model was trained that included both fMRI and behavior measures. The behavior measures encompassed quantitative sensory testing and questionnaires, including laser heat pain threshold, and cold pain threshold and tolerance, the Chinese versions of Fear of Pain Questionnaire, Pain Anxiety Symptoms Scale‐20, Pain Catastrophizing Scale, Pain Vigilance and Awareness Questionnaire, Beck Depression Inventory, Behavioral Activation and Behavioral Inhibition Scales, Revised Life Orientation Test, Pittsburgh Sleep Quality Index, Interpersonal Reactivity Index, Big Five Personality Test, and State‐Trait Anxiety Inventory (see supplemental data file 1 for details). As a first step to build this ensemble model, a behavior only model was trained apart from the fMRI only NIPS model. Since the number of features in the behavior only model was limited, the PCA step was omitted, and only standardized behavioral features and then input them to the LASSO algorithm. The optimal λ was selected from 100 values in [10^−^2, 102] by five‐fold cross‐validation, and turned out to be 0.196. The fMRI + behavior ensemble model was a stacking regression model.^[^ ^101^ ^]^ Specifically, the predictions from the fMRI model (LASSO‐PCR) and behavior model (LASSO) were used as features in a final multiple regression model. This stacking model had two tuning parameters λ_1_ and λ_2_ for the embedded fMRI model and behavior model respectively. Since all combinations of λ_1_ and λ_2_ need to be explored in a grid search, training stacking models was computationally intensive. Thus, 10 values log‐uniformly were only selected distributed in [10^−2^, 102] for both λ_1_ and λ_2_. Five‐fold cross‐validation led to the following optimal parameters: λ_1_ = λ_2_ = 0.215. Note that while this stacking model had better performance (Figure S9, Supporting Information), behavioral measures were not available in other datasets except for Datasets 1 and 2, resulting in the inability to test the generalizability of the fMRI + behavior model.

### Model Comparisons

To compare NIPS with the well‐established NPS^[^ ^46^ ^]^ and SIIPS,^[^ ^47^ ^]^ their performance was systematically examined, including generalizability, within‐individual pain prediction, and pain reduction prediction. In within‐individual pain prediction, single‐trial fMRI responses to painful stimuli were estimated with the Least‐Squares All approach,^[^ ^102^ ^]^ which treats every single trial as a separate regressor in general linear models. Dot products of the model weight and first‐level maps were taken as model predictions for NPS and SIIPS.

## Conflict of Interest

The authors declare no conflict of interest.

## Author Contributions

L.B.Z. and L.H. performed conceptualization, methodology, formal analysis, and visualization. H.J.Z., L.H., Z.X.W., and L.B.Z. performed investigation. L.B.Z. performed wrote the original draft. L.H. performed funding acquisition, project administration, and supervision. L.B.Z., X.J.L., H.J.Z., Z.X.W., Y.Z.K., Y.H.T., G.D.I., and L.H. performed wrote, reviewed, and edited the draft.

## Supporting information



Supporting Information

Supporting Information

Supporting Information

Supporting Information

## Data Availability

The data and code for all results are available on the Open Science Framework (https://osf.io/y2n34/).
