# Supplementary material for: A Replicable and Generalizable Neuroimaging‐Based Indicator of Pain Sensitivity Across Individuals
Source: Adv Sci (Weinh). 2025 Sep 5;12(44):e03373. doi: 10.1002/advs.202503373 (PMC12667548; doi:10.1002/advs.202503373)
Supplement: Supplementary file 1 — Supporting Information [file ADVS-12-e03373-s003.pdf]

Supporting Information

A Replicable and Generalizable Neuroimaging-based Indicator of Pain Sensitivity Across Individuals

Li-Bo Zhang[1,2,3], Xue-Jing Lu[1,2], Hui-Juan Zhang[1,2], Zhao-Xing Wei[1,2], Ya-Zhuo Kong[1,2], Yi-Heng Tu[1,2], Gian Domenico Iannetti[3,4], Li Hu[1,2,]*

[1]State Key Laboratory of Cognitive Science and Mental Health, Institute of Psychology, Chinese Academy of Sciences, 100101, Beijing, China;

[2]Department of Psychology, University of Chinese Academy of Sciences, 100049, Beijing, China;

[3]Neuroscience and Behaviour Laboratory, Italian Institute of Technology, 00161, Rome, Italy;

[4]Department of Neuroscience, Physiology and Pharmacology, University College London, WC1E 6BT, London, UK.

[*]Corresponding author

Supplemental figures

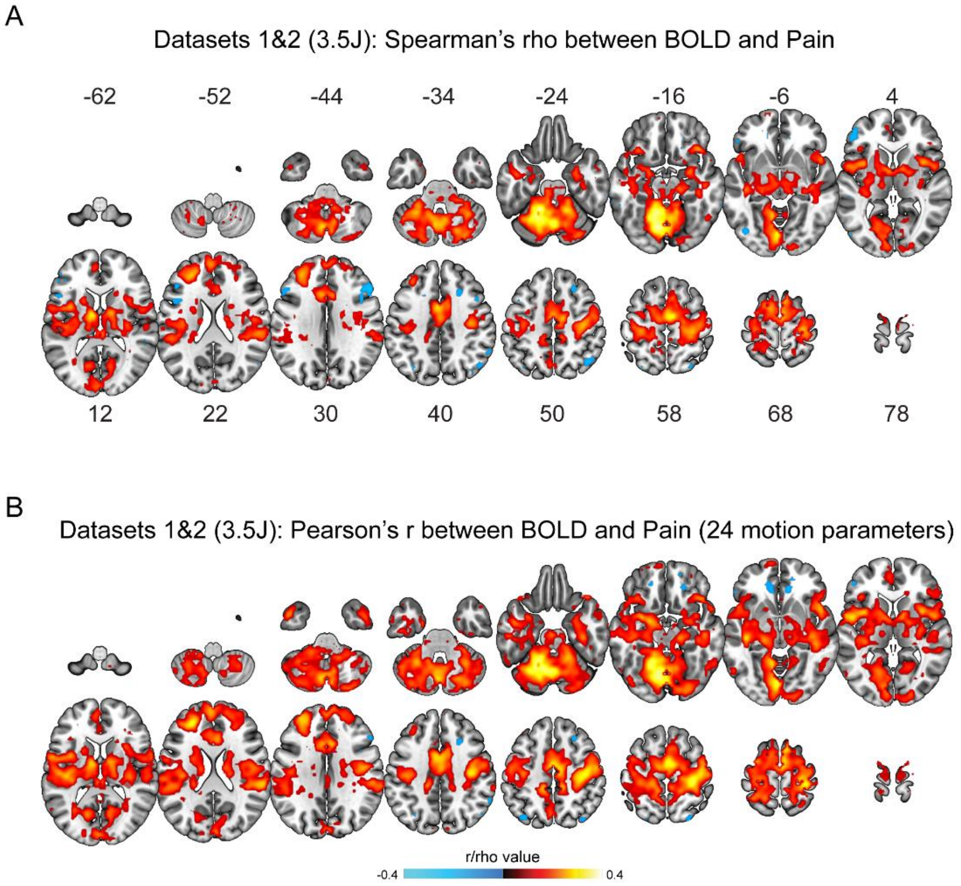

Figure S1. Robustness of the correlation between BOLD responses and pain sensitivity in Datasets 1&2. (A) Nonparametric Spearman correlation between nociceptive-evoked BOLD responses and pain sensitivity in Datasets 1&2. Nonparametric correlation revealed similar correlational patterns as parametric correlation. **(B)** Correlational results when head motion was controlled with 24 parameters in Datasets 1&2. Strict control of head motion did not substantially change the correlational patterns between BOLD responses and pain sensitivity.

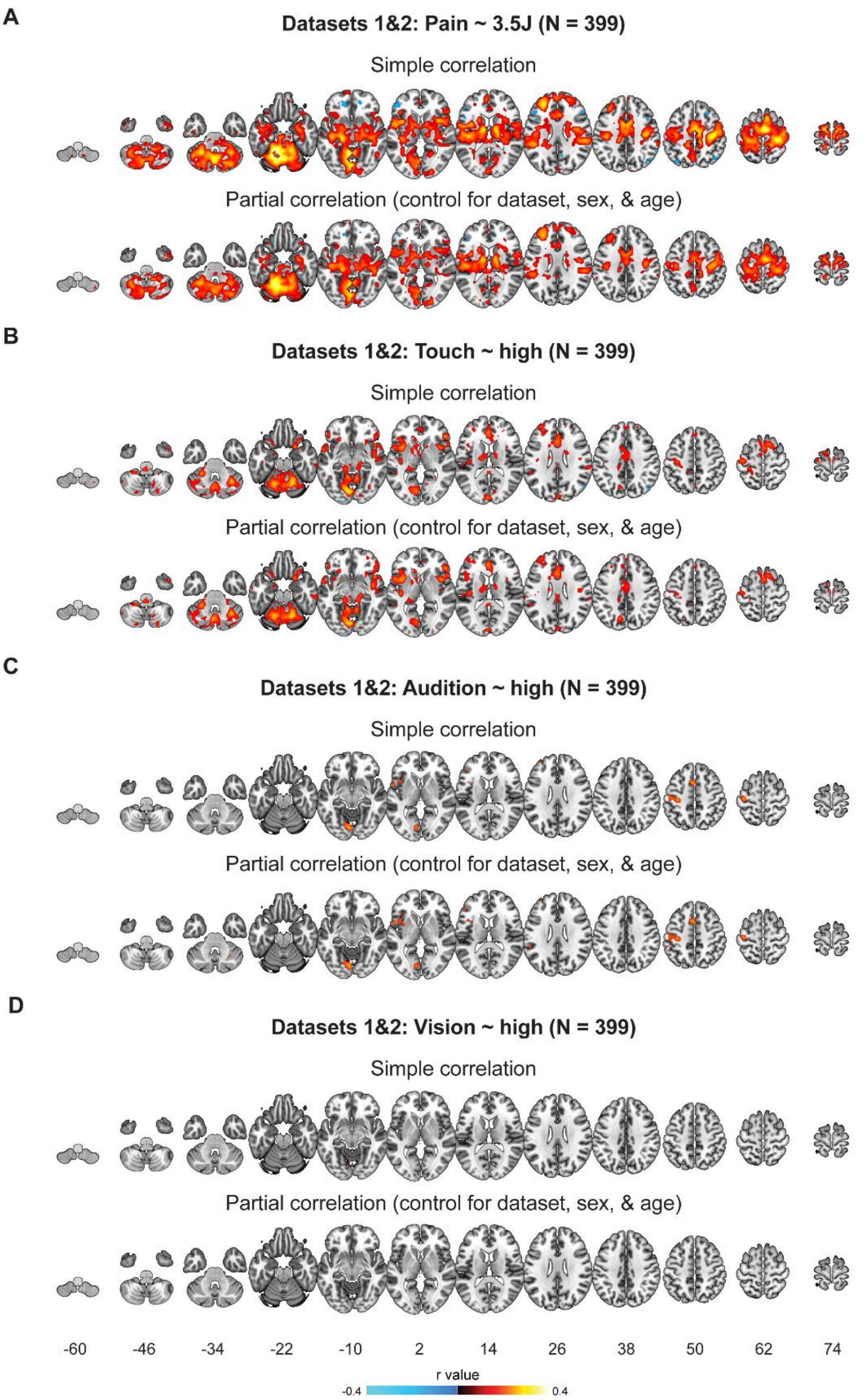

Figure S2. Comparisons of results between simple correlation and partial correlation results for different sensory modalities in Datasets 1&2. Simple correlation results were identical to those reported in Figures 2 and 4. Partial correlation results were controlled for dataset identity (dummy coded as 0 and 1), sex (dummy coded as 0 and 1 for males and females), and age. Controlling for dataset, sex, and age slightly decreased the magnitude of correlations between fMRI response and sensory sensitivities, but the general pattern remained the same.

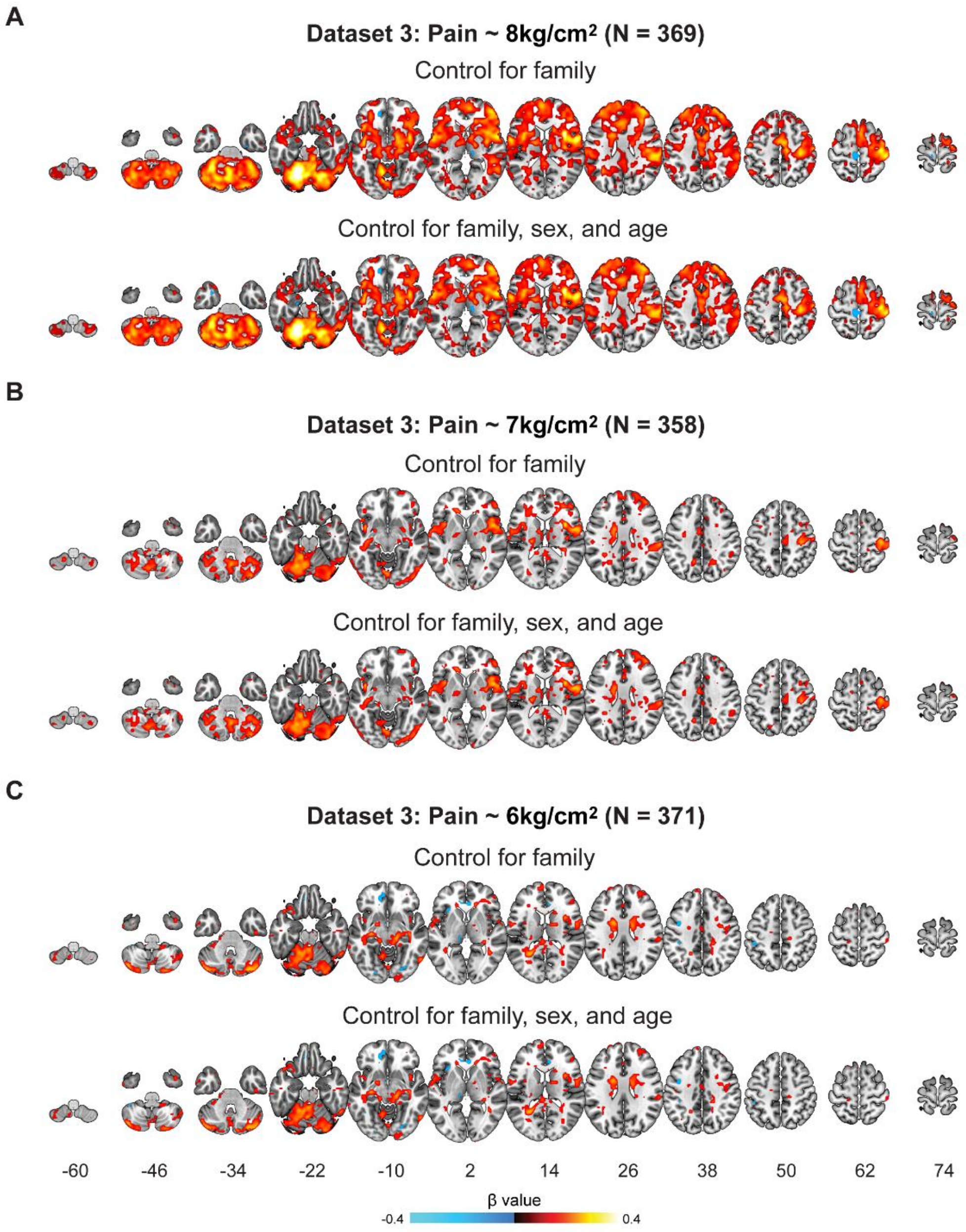

Figure S3. Comparisons of results before and after controlling for sex and age in Dataset 3. Further control over sex and age had no substantial effect on the associations between fMRI responses and mechanical pain sensitivity.

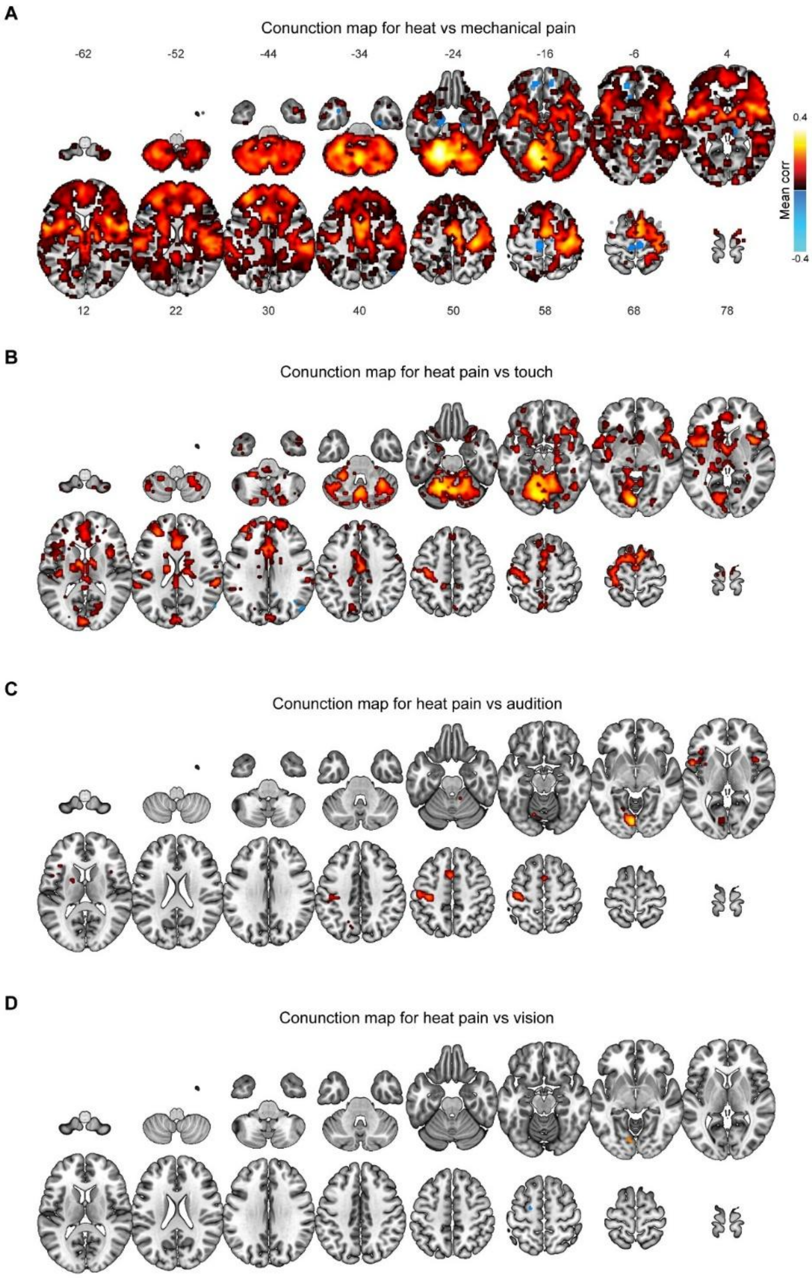

Figure S4. Conjunction maps for pain and nonpain correlation maps. Heat pain is the 3.5J condition in Datasets 1&2; mechanical pain in the 8kg/cm^2 condition in Dataset 3; touch, audition, and vision are all high intensity conditions in Datasets 1&2. Mean r values across the two conditions are plotted.

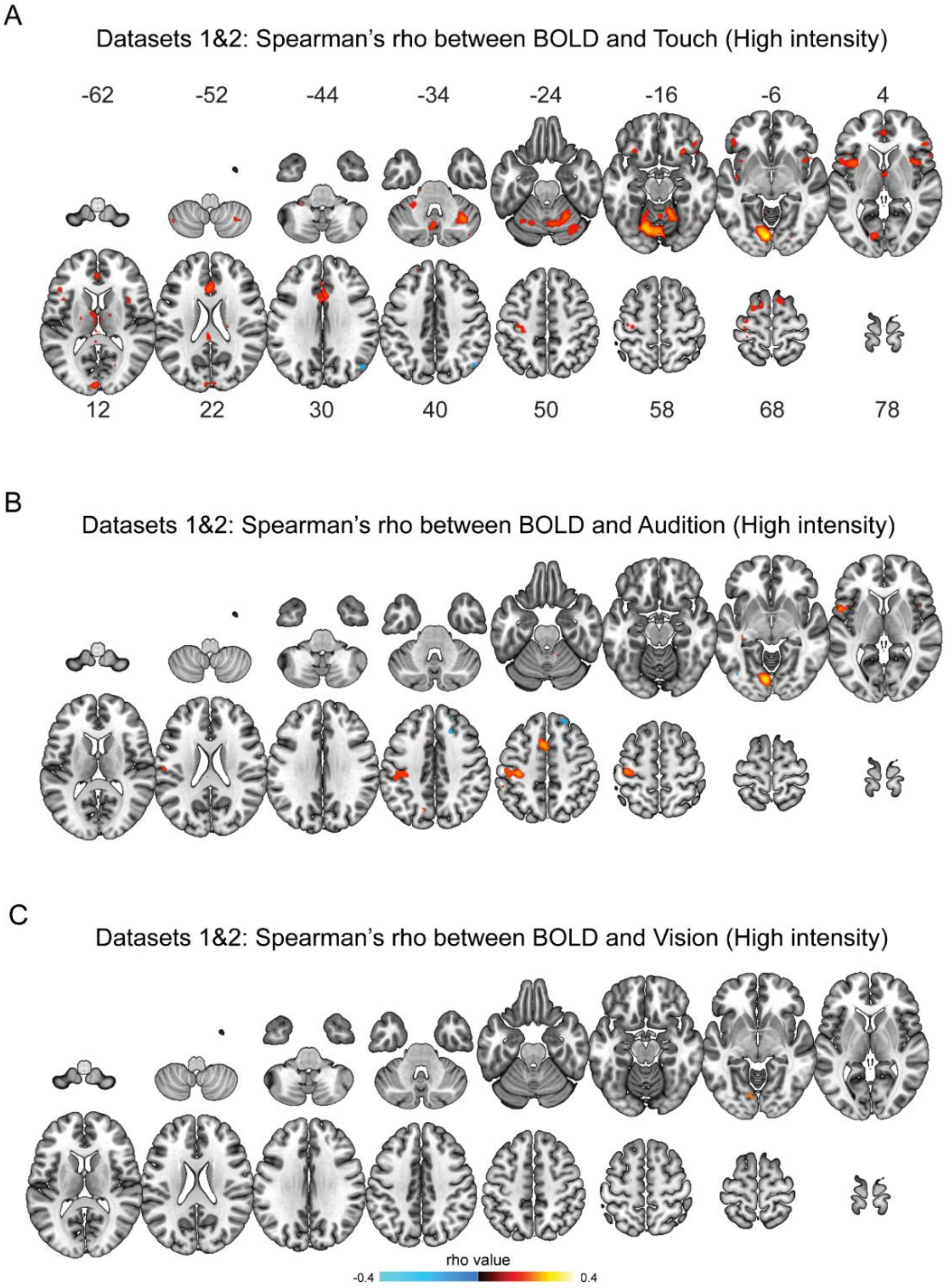

Figure S5. Robustness of the correlation between BOLD responses and nonpain sensitivity in the high intensity condition in Datasets 1&2. Nonparametric Spearman correlation between BOLD responses and tactile (A), auditory (B), and visual (C) sensitivity in the high intensity condition in Datasets 1&2. Nonparametric correlation revealed similar correlational patterns as parametric correlation.

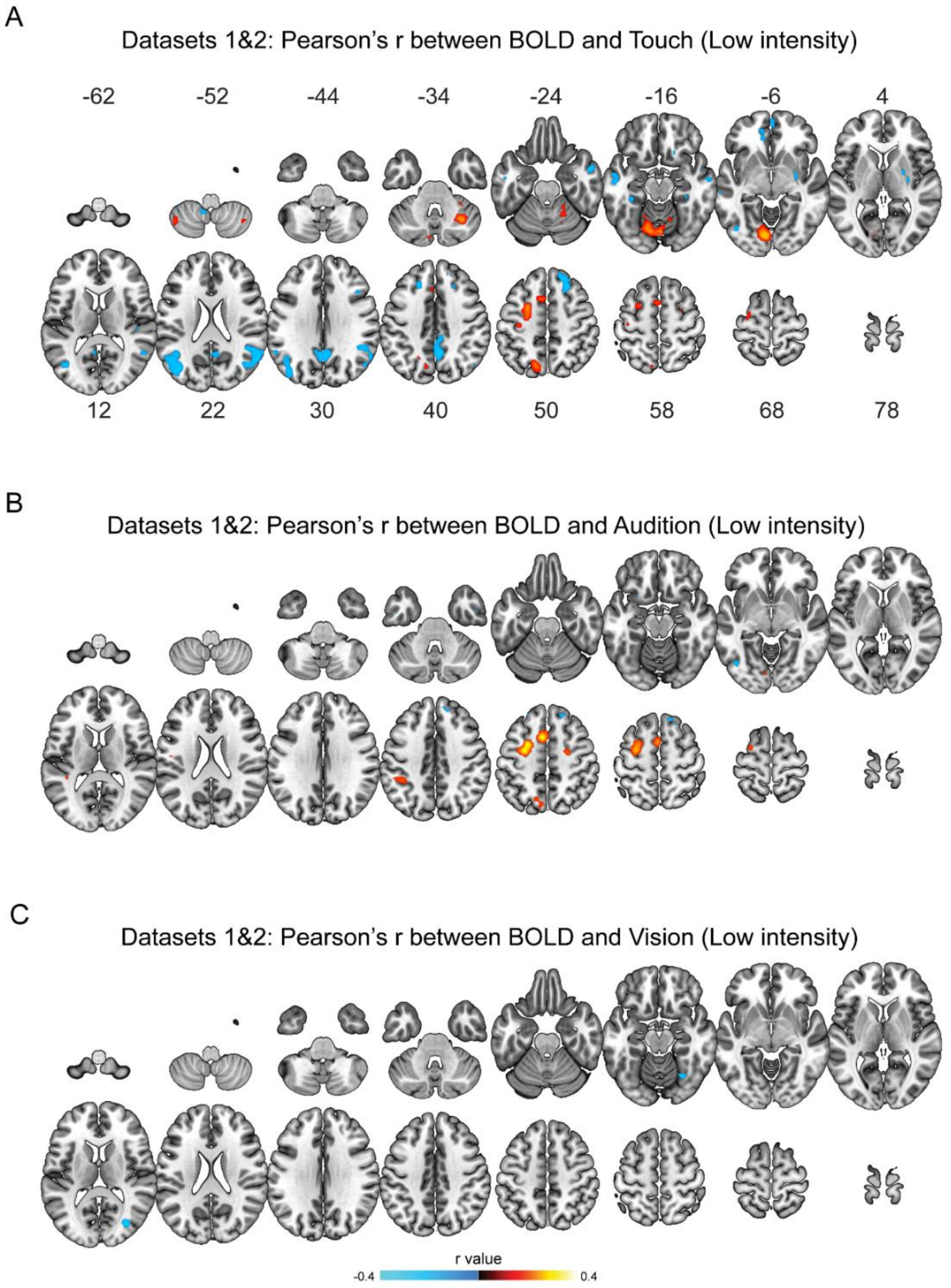

Figure S6. Correlation between BOLD responses and nonpain sensitivity in the low intensity condition in Datasets 1&2. Pearson correlation between BOLD responses and tactile (A), auditory (B), and visual (C) sensitivity in the low intensity condition in Datasets 1&2. Correlational patterns in the low intensity condition differed from those in the high intensity condition, suggesting an influence of stimulus intensity.

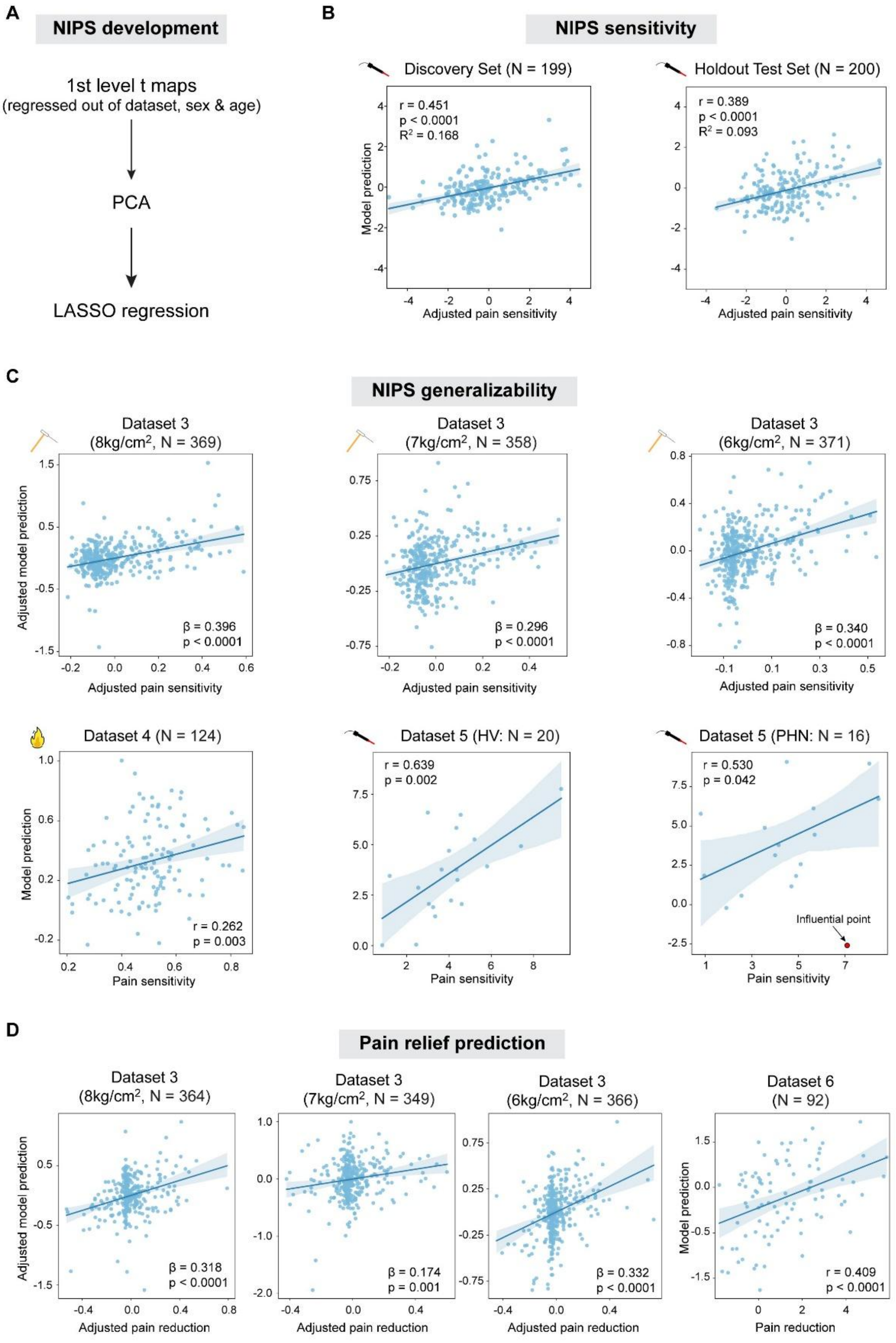

Figure S7. Performance of multivariate model after regressing out control variables. The model was built with fMRI responses and pain ratings that were adjusted by regressing out dataset, sex, and age. In panel B (Datasets 1&2), "adjusted pain sensitivity" means that average pain ratings were adjusted for dataset, sex, and age. In panels C&D (Dataset 3), "adjusted" means that fixed effects of sex and age, and random effects of family were adjusted. β in panels C&D is the standardized coefficient in

mixed effects models. Note that real and predicted values in Datasets 4~6 were not adjusted since dataset heterogeneity and dependent observations were not present in these datasets. Influential points in Dataset 5 were identified according to Cook's distance > 0.5, and removed to obtain robust results. HV: healthy volunteers; PHN: postherpetic neuralgia patients.

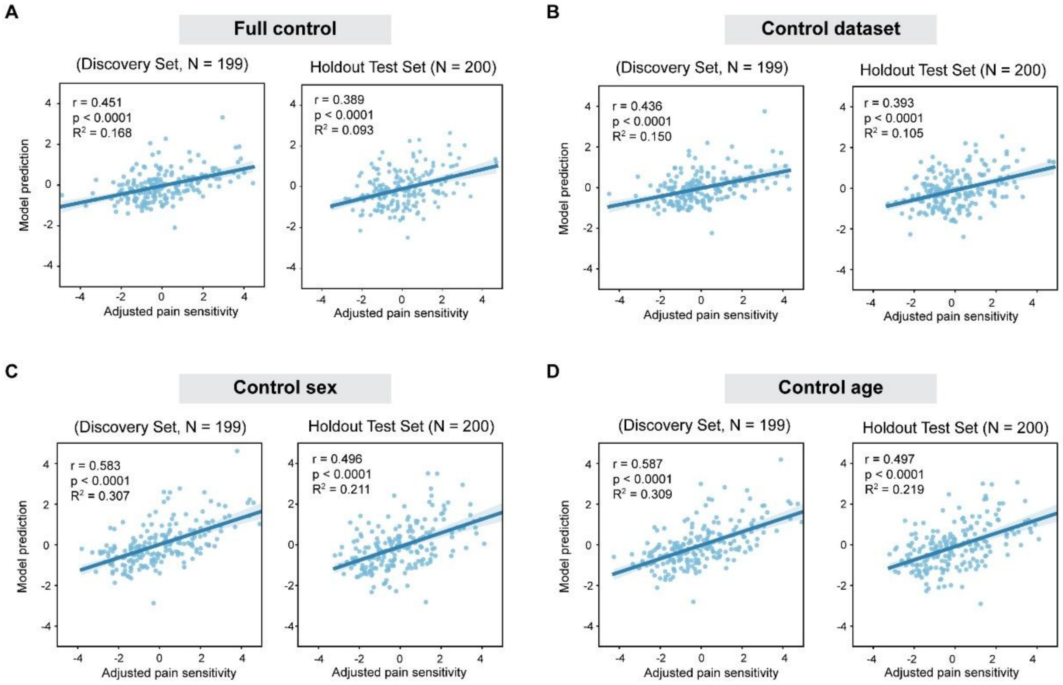

Figure S8. Contribution of covariates to dataset heterogeneity. The model was built with fMRI responses and pain ratings that were adjusted by regressing out dataset identity, sex, or age. "Adjusted pain sensitivity" means that average pain ratings were adjusted for corresponding variable(s).

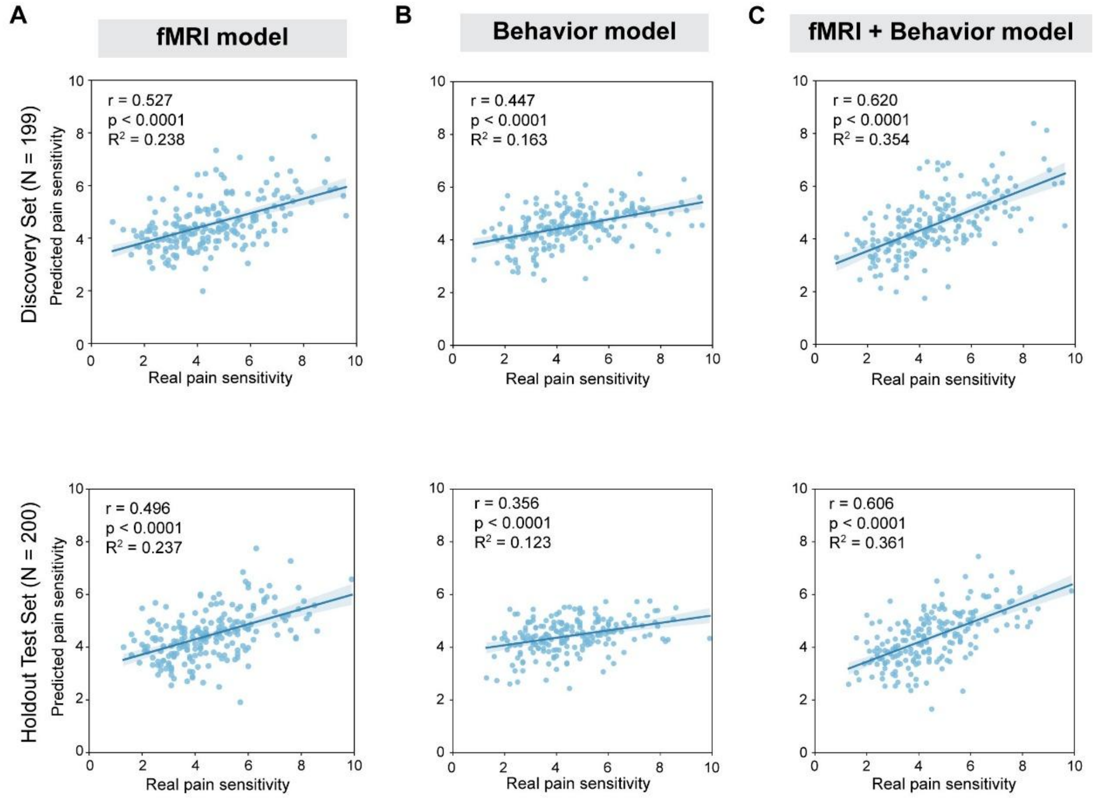

Figure S9. Performance of fMRI only, behavior only, and fMRI + behavior models. Compared with fMRI only (A) and behavior only (B) models, a composite model including both fMRI responses and behavioral measures had better performance.

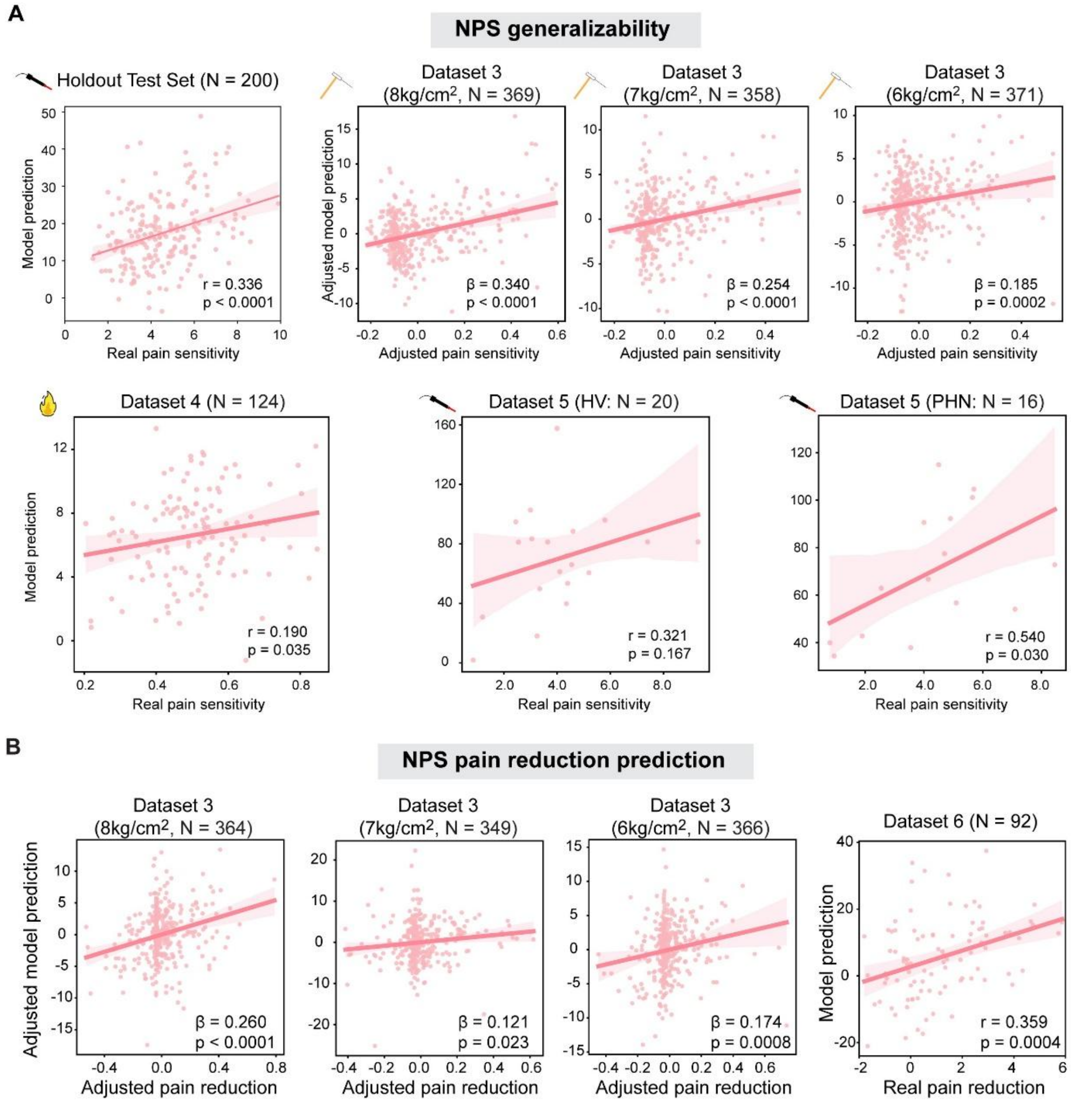

Figure S10. Performance of NPS. "Adjusted pain sensitivity" means that average pain ratings were adjusted for dataset identity. "Adjusted" means random effects of family were adjusted. β in panels B&C is the standardized coefficient in mixed effects models.

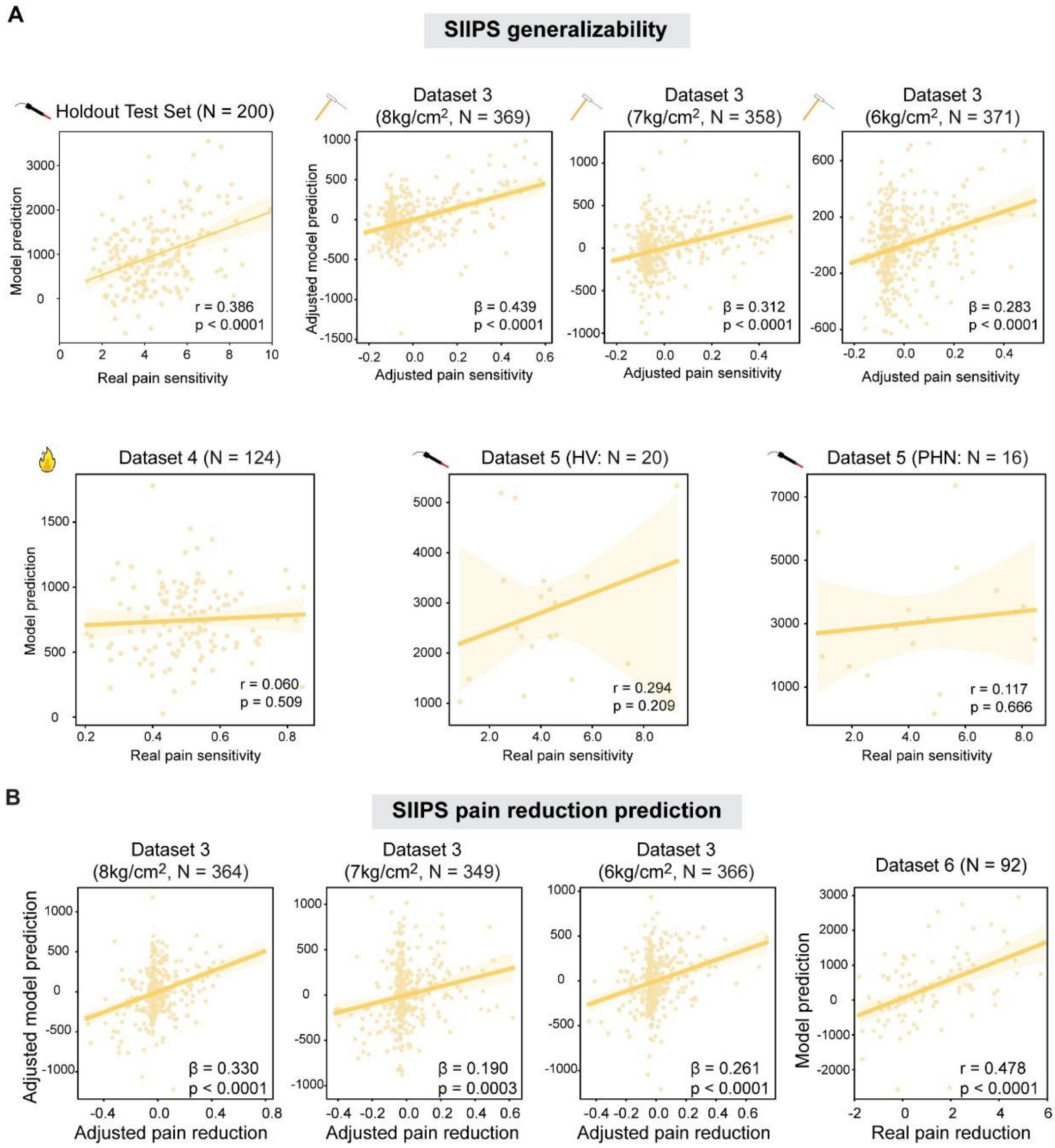

Figure S11. Performance of SIIPS. "Adjusted pain sensitivity" means that average pain ratings were adjusted for dataset identity. "Adjusted" means random effects of family were adjusted. β in panels B&C is the standardized coefficient in mixed effects models.

Supplemental tables

Table S1. Dataset summary

	**Dataset 1**	**Dataset 2**	**Dataset 3**	**Dataset 4**	**Dataset 5 (Patient)**	**Dataset 5 (Healthy)**	**Dataset 6**
N	212*	187*	395	124	16	20	92
Sex (M:F)	76:135	103:83	164:231	63:61	5:11	8:12	42:50
Age (years)	21.5 ± 4.2	21.0 ± 3.3	35.4 ± 2.6	22.2 ± 2.7	65.8 ± 7.0	61.6 ± 8.2	21.9 ± 3.2
Intervention	/	/	Placebo, control	/	/	/	c-TENS, a-TENS, sham
Pain stimulus	Laser heat	Laser heat	Mechanical, contact heat#	Contact heat	Laser heat	Laser heat	Contact heat
Nonpain stimulus	Electro-tactile, auditory, visual	Electro-tactile, auditory, visual	/	/	/	/	/
Intensity level	2	2	3	6&	1	1	1$
Rating scale	0-10	0-10	0-1	0-1	0-10	0-10	0-10

Note: *: One participant did not provide demographic information. #: Contact heat pain data were not analyzed in this study. &: Data were only available for one intensity level. $: Perceived intensity ratings were fixed, not physical intensity.

Table S2. Functional MRI acquisition parameters

Parameters	Datasets 1&2	Datasets 3&4	Dataset 5	Dataset 6
MRI scanner	GE MR 750	Siemens Prisma	GE MR 750	Siemens Prisma
Magnetic strength	3T	3T	3T	3T
Field of view (mm)	192	220	220	192
Number of slices	43	56	43	70
Slice thickness (mm)	3	2.7	3	4
TR (ms)	2000	460	2000	2680
TE (ms)	29	27.2	30	27
Flip angle (deg)	90	44	70	80
Slice order	Ascending (interleaved)	Ascending (interleaved)	Ascending (interleaved)	Ascending (interleaved)

Table S3. Structural MRI acquisition parameters

Parameters	Datasets 1&2	Datasets 3	Datasets 4	Dataset 5	Dataset 6
MRI scanner	GE MR 750	Siemens Prisma	Siemens Prisma	GE MR 750	Siemens Prisma
Magnetic strength	3T	3T	3T	3T	3T
Field of view (mm)	256	256	256	256	288
Slice thickness (mm)	1	0.8	0.7	1	1
TR (ms)	6.896	2000	2400	6.9	2300
TE (ms)	2.99	2.11	2.34	2.9	2.28
Flip angle (deg)	8	8	8	8	8
Slice order	Ascending (interleaved)	Ascending (interleaved)	Ascending (interleaved)	Ascending (interleaved)	Ascending (interleaved)

Table S4. Intensity ratings in different datasets

Datasets	Condition	Mean	SD	Max	Min
Dataset 1	Pain (3.5J)	5.294	1.637	9.900	0.800
	Pain (3.0J)	4.263	1.534	8.500	0.600
	Touch (High)	4.943	1.524	9.300	1.200
	Touch (Low)	3.846	1.577	8.800	0.800
	Audition (High)	4.614	1.544	9.300	1.900
	Audition (Low)	2.873	1.217	7.700	0.700
	Vision (High)	6.030	1.512	9.500	2.300
	Vision (Low)	3.463	1.128	7.400	1.100
Dataset 2	Pain (4.0J)	4.547	1.480	8.800	1.200
	Pain (3.5J)	3.675	1.367	8.500	1.200
	Touch (High)	4.572	1.718	9.700	0.200
	Touch (Low)	3.519	1.591	7.800	0.200
	Audition (High)	4.235	1.646	8.700	1.000
	Audition (Low)	2.672	1.323	7.100	0.400
	Vision (High)	5.627	1.525	9.300	1.800
	Vision (Low)	3.093	1.082	7.100	1.100
Dataset 3	Control (8kg/cm^2)	0.150	0.193	0.900	0.000
	Control (7kg/cm^2)	0.131	0.175	0.873	0.000
	Control (6kg/cm^2)	0.123	0.160	0.900	0.000
	Placebo (8kg/cm^2)	0.113	0.175	0.886	0.000
	Placebo (7kg/cm^2)	0.092	1.148	0.816	0.000
	Placebo (6kg/cm^2)	0.085	0.129	0.826	0.000
Dataset 4	Pain (47.5°C)	0.500	0.130	0.847	0.204
Dataset 5	Patient (3.5J)	4.503	2.257	8.450	0.800
	Healthy (3.5J)	4.070	1.927	9.300	0.850
Dataset 6	Pre-treatment (Pain)	6.591	1.045	9.130	4.530
	Post-treatment (Pain)	5.431	1.869	9.070	1.270

Table S5. Correlation between brain activations and pain ratings in ROIs in Datasets 1&2

Datasets	ROIs	r	P(raw)	P(FDR)
Datasets 1&2 (3.5J)	S1	0.248	<0.001	<0.001
	S2	0.273	<0.001	<0.001
	ACC	0.241	<0.001	<0.001
	insula	0.190	<0.001	<0.001
	thalamus	0.222	<0.001	<0.001
	cerebellum	0.338	<0.001	<0.001
Dataset 1 (3.0J)	S1	0.194	0.005	0.005
	S2	0.306	<0.001	<0.001
	ACC	0.220	0.001	0.001
	insula	0.253	<0.001	<0.001
	thalamus	0.270	<0.001	<0.001
	cerebellum	0.332	<0.001	<0.001
Dataset 2 (4.0J)	S1	0.211	0.004	0.008
	S2	0.228	0.002	0.005
	ACC	0.139	0.058	0.058
	insula	0.203	0.005	0.008
	thalamus	0.141	0.054	0.058
	cerebellum	0.229	0.002	0.005

Note: Cells highlighted in red show significant correlation after FDR correction.

Table S6. Correlation between brain activations and pain ratings in ROIs in Dataset 3

Conditions	ROIs	β	P(raw)	P(FDR)
8kg/cm^2	S1	0.151	0.003	0.004
	S2	0.149	0.004	0.004
	ACC	0.230	<0.001	<0.001
	insula	0.284	<0.001	<0.001
	thalamus	0.149	0.003	0.004
	cerebellum	0.315	<0.001	<0.001
7kg/cm^2	S1	0.080	0.121	0.121
	S2	0.118	0.023	0.027
	ACC	0.142	0.006	0.012
	insula	0.189	<0.001	<0.001
	thalamus	0.123	0.016	0.024
	cerebellum	0.208	<0.001	<0.001
6kg/cm^2	S1	0.025	0.619	0.691
	S2	0.051	0.319	0.479
	ACC	0.080	0.117	0.235
	insula	0.104	0.039	0.198
	thalamus	0.020	0.691	0.691
	cerebellum	0.209	<0.001	<0.001

Note: β is the standardized coefficient in mixed effects models that take into account observation dependence within families. Cells highlighted in red show significant coefficients after FDR correction.
